# Replicating PET Hydrolytic Activity by Positioning Active Sites with Smaller Synthetic Protein Scaffolds

**DOI:** 10.1002/advs.202500859

**Published:** 2025-03-16

**Authors:** Yujing Ding, Shanshan Zhang, Xian Kong, Henry Hess, Yifei Zhang

**Affiliations:** ^1^ State Key Laboratory of Chemical Resources Engineering Beijing University of Chemical Technology Beijing 100029 P. R. China; ^2^ Beijing Advanced Innovation Center for Soft Matter Science and Engineering Beijing University of Chemical Technology Beijing 100029 P. R. China; ^3^ South China Advanced Institute for Soft Matter Science and Technology Guangdong Provincial Key Laboratory of Functional and Intelligent Hybrid Materials and Devices School of Emergent Soft Matter South China University of Technology Guangzhou 510640 P. R. China; ^4^ Department of Biomedical Engineering Columbia University 351L Engineering Terrace, 1210 Amsterdam Avenue New York NY 10027 USA

**Keywords:** artificial enzyme, computational enzyme design, isoenzyme diversity, new‐to‐nature PET hydrolase, small enzyme

## Abstract

Evolutionary constraints significantly limit the diversity of naturally occurring enzymes, thereby reducing the sequence repertoire available for enzyme discovery and engineering. Recent breakthroughs in protein structure prediction and *de novo* design, powered by artificial intelligence, now enable to create enzymes with desired functions without solely relying on traditional genome mining. Here, a computational strategy is demonstrated for creating new‐to‐nature polyethylene terephthalate hydrolases (PET hydrolases) by leveraging the known catalytic mechanisms and implementing multiple deep learning algorithms and molecular computations. This strategy includes the extraction of functional motifs from a template enzyme (here leaf‐branch compost cutinase, LCC, is used), regeneration of new protein sequences, computational screening, experimental validation, and sequence refinement. PET hydrolytic activity is successfully replicated with designer enzymes that are at least 30% shorter in sequence length than LCC. Among them, *Rs*PETase1 stands out due to its robust expressibility. It exhibits comparable catalytic efficiency (*k*_cat_/*K*_m_) to LCC and considerable thermostability with a melting temperature of 56 °C, despite sharing only 34% sequence similarity with LCC. This work suggests that enzyme diversity can be expanded by recapitulating functional motifs with computationally built protein scaffolds, thus generating opportunities to acquire highly active and robust enzymes that do not exist in nature.

## Graphical abstract

A workflow is proposed for the computational design of new‐to‐nature PET hydrolases by remodeling the protein scaffold. Starting from the functional motifs extracted from a template enzyme, designer enzymes are generated following four steps: scaffold remodeling, computational screening, experimental validation, and sequence refinement. This strategy can increase the diversity of isoenzymes without relying on genome mining.

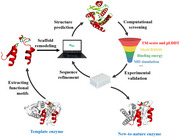

## 1 Introduction

Enzymes are essential workhorse molecules of both living organisms and biochemical industries due to their powerful and sophisticated catalytic functions.^[^ ^1^, ^2^, ^3^ ^]^ The activity of an enzyme is often due to a small number of amino acid residues that are arranged in a defined 3D configuration as a result of protein folding.^[^ ^4^ ^]^ This active site is surrounded by a large number of amino acids that form a protein scaffold, which holds the catalytic motifs in specific geometries and influences enzyme activity through its intrinsic protein dynamics. Similar catalytic functions are often found in enzymes that evolved independently with similar functional motifs from distinct ancestral proteins (examples of convergent evolution^[^ ^5^ ^]^), demonstrating that the protein scaffold can be varied. On the basis of this insight, past efforts of *de novo* design of enzymes have focused on the transplantation of functional motifs onto a foreign protein scaffold. Classic cases are the computational design of retro‐aldolases^[^ ^6^ ^]^ and Kemp eliminase.^[^ ^7^ ^]^ A very recent advancement is the engineering of Fragaceatoxin C nanopores for hydrolyzing nano‐sized polyethylene terephthalate (PET).^[^ ^8^ ^]^ However, this strategy relies on the availability of existing protein scaffolds with a certain level of structural similarity to the desired functional motifs.^[^ ^9^ ^]^ Furthermore, the introduction of new residues onto a native protein backbone may lead to unexpected geometrical variations in the active site.^[^ ^10^ ^]^ The limited availability of suitable scaffolds and the complexity of protein folding constrain our ability to design *de novo* enzymes.^[^ ^11^ ^]^

The rapid developments of artificial intelligence (AI) in protein science open up new opportunities to address these limitations. The deep learning program AlphaFold can predict the protein structure with atomic‐level accuracy based solely on the primary amino acid sequence.^[^ ^12^, ^13^, ^14^ ^]^ Two deep‐learning methods, constrained hallucination and inpainting, can generate artificial protein backbones to scaffold pre‐specified functional sites.^[^ ^15^ ^]^ Alternatively, a family‐wide hallucination approach can generate large number of new idealized protein scaffolds for accommodating target substrates.^[^ ^16^ ^]^ A protein language model called ProGen was trained to produce protein sequences with a predictable function across diverse protein families.^[^ ^17^ ^]^ These advancements substantially improve the success rate for the design of new‐to‐nature enzymes.

The diversity of naturally occurring enzymes is constrained during evolution by the ancestral protein sequences and the need to maintain the homeostasis of a living system, which consequently restricts the sources of enzymes for industrial applications. *De novo* enzyme design provides a way to create the diversity of enzymes with desired activities without relying on conventional enzyme mining. For instance, Yeh et al. designed artificial luciferases with a smaller size that exhibit high activity and exceptional thermostability, outperforming their native counterparts.^[^ ^16^ ^]^ This success encourages efforts to reduce the size of enzymes. However, previous attempts to achieve this have failed to produce catalytically active proteins,^[^ ^18^ ^]^ highlighting the challenges of replicating enzyme activity without a deep understanding of the intricate interactions between the protein scaffold and the substrate.

Recently, researchers have made significant attempts to identify potential PET hydrolytic enzymes from the environment to address the global plastic pollution.^[^ ^19^, ^20^ ^]^ These efforts have resulted in dozens of different enzymes with known activity against PET.^[^ ^21^ ^]^ Among them, the most promising ones for industrial PET recycling are the mutants engineered from *Ideonella sakaiensis* PETase (*Is*PETase, 290 amino acids, *M*_w_ = 30.25 kDa)^[^ ^20^, ^22^ ^]^ and leaf‐branch compost cutinase (LCC, 258 amino acids, *M*_w_ = 27.78 kDa).^[^ ^23^ ^]^ These PET hydrolases share a conserved catalytic triad of serine (Ser)‐histidine (His)‐aspartate (Asp) residues borne by protein backbones encoded by distinctly different amino acid sequences. This inspired us to design new‐to‐nature PET hydrolases by re‐scaffolding the catalytic triad with AI‐generated protein scaffolds.

Building on the demonstration that the inpainting method has the ability to generate new proteins with binding activity inherent in functional motifs,^[^ ^15^ ^]^ we employed inpainting to generate new and shorter sequences encoding protein scaffolds to support the catalytic triad of LCC and some adjacent residues. Taking advantage of the protein structures predicted by ColabFold (an online tool integrating AlphaFold2 with the fast homology search of MMseqs2),^[^ ^24^ ^]^ we conducted in silico screening for the virtual designer enzymes that are putatively capable of hydrolyzing PET. We evaluated the expression and function of ten designer enzymes obtained from the screening, and found that 8 enzymes were capable of hydrolyzing PET nanoparticles (PET‐NPs, ≈100 nm in diameter). The difficulty in expression and absence of hydrolytic activity toward PET microparticles (tens of micrometers in diameter) led us to revisit and refine the sequences by iterative computation. Two designer enzymes were obtained after structure revision that exhibit PET hydrolytic activity but have low expressibility. After sequence refinement using ProteinMPNN,^[^ ^25^ ^]^ we created an expressible smaller artificial PET hydrolase with catalytic efficiency (*k*_cat_/*K*_m_) comparable to its template LCC. This designer enzyme is more compact than LCC and has fewer α‐helices and β‐sheets in its fold, suggesting that the natural enzymes can be miniaturized through our method to reduce potentially redundant protein structures. The successful creation of a synthetic scaffold for a known catalytic mechanism demonstrates the effectiveness of deep learning methods in the construction of designer enzymes, enabling the computational exploration of the protein universe beyond conventional genome mining to craft novel enzymes with targeted activities.

## 2 Results and Discussion

### 2.1 A General Workflow for the Computational Design of PET Hydrolases

**Figure 1:**
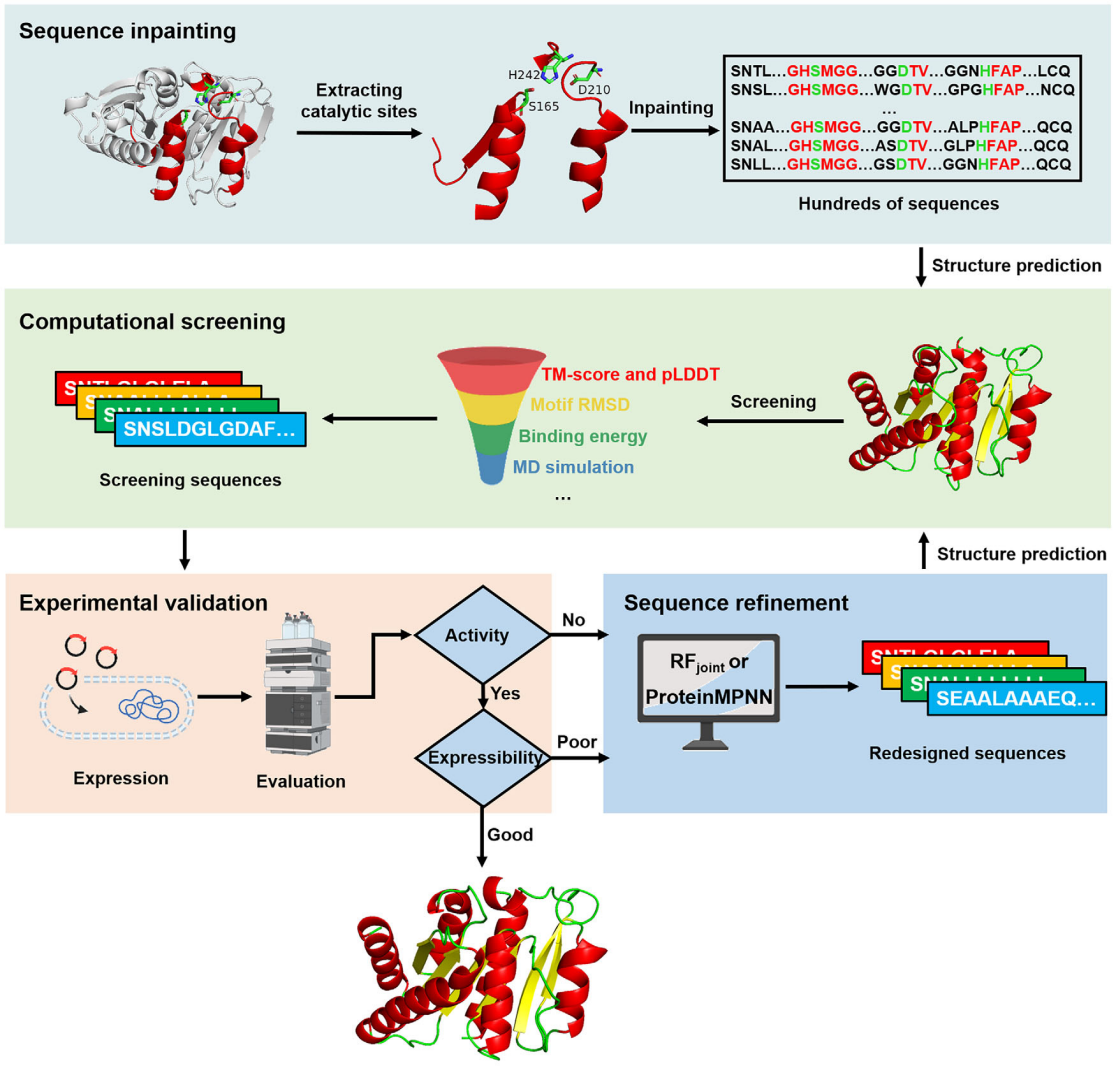
A workflow for the computational design of new‐to‐nature PET hydrolases by remodeling the protein scaffold. The workflow includes four steps: sequence inpainting, in which the functional motifs are extracted from a template enzyme and then the missing sequences are completed by inpainting; computational screening, in which the newly generated sequences are screened computationally based on a set of physicochemical criteria; experimental validation, in which the filtered sequences are examined in terms of expression and expected activity; sequence refinement, in which flaws in designs are revised. Iterative implementation of the sequence refinement, computational screening, and experimental validation steps effectively improve the quality of designs.

Currently known PET hydrolases, including LCC and *Is*PETase, possess the same catalytic triad of Ser‐His‐Asp. The specific spatial arrangement of these key amino acids creates the ester hydrolytic functionality. At the same time the amino acids around the catalytic triad form a substrate‐binding groove that helps to stabilize the reaction transition state via electrostatic interactions and hydrogen bonding.^[^ ^26^, ^27^ ^]^ Since LCC and its variations are more catalytically active and thermally stable than *Is*PETase, we attempt to generate new‐to‐nature PET hydrolases by re‐scaffolding the functional motifs of LCC. The LCC variants, including LCC^ICCG^, LCC^WCCM^, and LCC^WCCG^, exhibit identical specific activities but higher melting temperatures,^[^ ^28^ ^]^ indicating that these mutations primarily influence protein stability rather than the transition states. Therefore, our design begins with the catalytic motifs of the wild‐type LCC. Moreover, as LCC is the smallest PET hydrolase yet discovered, we sought to design an even smaller designer counterpart. The workflow we follow to generate designer enzymes is shown in a simplified version in **Figure** **1**. We first identify the active sites of LCC based on molecular docking, and then extract the catalytic motifs together with adjacent pieces of secondary structures. We use RF_joint_‐guided inpainting to fill in additional sequences encoding new protein structures to scaffold the extracted structures.^[^ ^15^ ^]^ We then use ColabFold to predict the 3D protein structures of the generated sequences. The virtual structures with high prediction certainty are selected for further computational screening. The screening considers the motif root mean square deviation (RMSD) between the backbone atoms of the retained LCC segments and those from the virtual enzymes, the binding energy with a substrate molecule, and the time‐dependent global Cα‐RMSD of the virtual enzymes by Molecular Dynamics (MD) simulation. Designed sequences often face challenges with expression and folding. We experimentally demonstrate that problematic sequences generated by inpainting can be revised by executing the RF_joint_ step multiple times and the active but difficult‐to‐express sequences can be rescued by using ProteinMPNN. By iteratively performing the sequence inpainting, computational screening, experimental validation and sequence refinement, we can obtain active and expressible new enzymes whose sequences and structures are very different from the template. Detailed considerations for sequence design, screening, refinement, along with the associated challenges and solutions, are discussed in the context. We believe this workflow can, in principle, be adapted for the de novo design of other enzymes with known catalytic motifs.

### 2.2 In Silico Generation and Screening of Virtual PET Hydrolases

**Figure 2:**
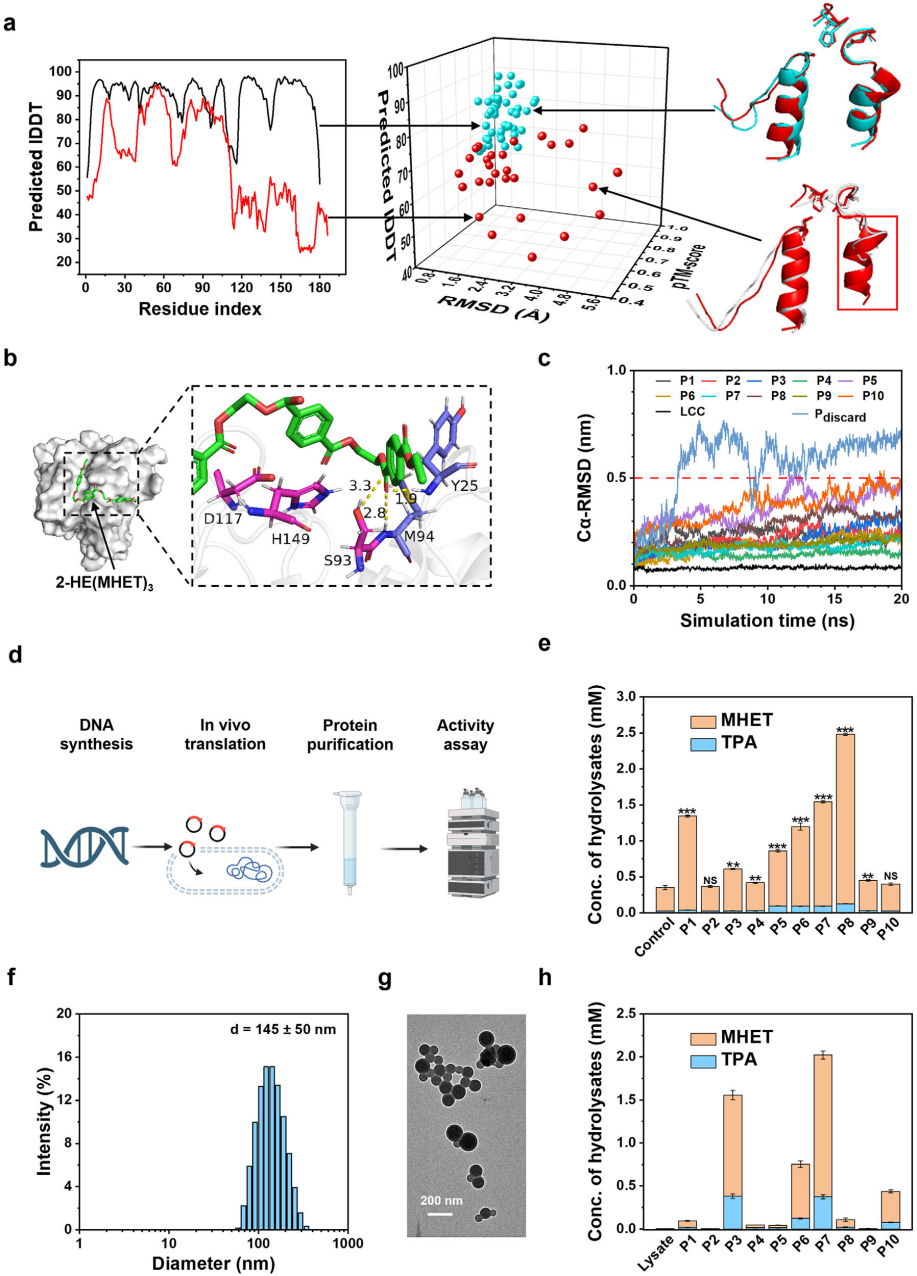
Typical results of the computational screening. a) A 3D scatter plot showing the AlphaFold pLDDT, pTM‐score and the motif RMSD of the computationally generated virtual enzymes. Examples of virtual enzymes with qualified pLDTT (black curve) and unqualified pLDTT (red curve) are shown on the left. Examples of virtual enzymes with the motif RMSD of 2.00 Å (blue) and 4.82 Å (white) compared to the retained motifs from LCC (red) are shown on the right. b) Molecular docking of 2‐HE(MHET)_3_ (green stick model) in a virtual enzyme (here P1 is used as an example). 2‐HE(MHET)_3_ interacts with the catalytic triad (magenta stick model) and the oxyanion hole potentially formed by the NH groups of Y25 and M94. c) Time‐course RMSD of Cα of representative virtual enzymes and LCC assessed by MD simulations. d) Procedures for the expression, purification and activity assay of the designer enzymes. e) The hydrolysis of BHET by the eluted protein fractions from a Ni‐NTA affinity column. The reaction system contained 6.0 µg mL^−1^ proteins and 1.0 mg mL^−1^ BHET and was incubated at 25 °C for 24 h. Control represents the self‐hydrolysis of BHET. A t‐test was used for statistical analysis, *p* < 0.001 (***), *p* < 0.01 (**), *p* < 0.05 (*), and NS, no significant difference. f) DLS analysis of the PET‐NPs. g) A TEM image of the PET‐NPs. h) The hydrolysis of 1.0 mg mL^−1^ PET‐NPs by the eluted protein fractions (6.0 µg mL^−1^) from a Ni‐NTA affinity column at 50 °C for 24 h. The cell lysate of *E. coli* carrying empty plasmids was used a benchmark. Error bars represent the standard deviations of three measurements.

To guarantee the success rate of the computational design and minimize the search space, we retained three disconnected LCC sequence segments so that each segment encodes a structural motif containing a key residue of the indispensable catalytic triad, S165/D210/H242. The retained sequences consist of 44 amino acid residues in total, accounting for 17% of the full‐length LCC. We then generated complementary protein structures to recapitulate the retained motifs in place by using a RF_joint_‐guided inpainting approach. We intentionally restricted the length of the in silico generated sequences to be shorter than LCC (Table S1, Supporting Information). The inpainting step generated hundreds of distinct protein sequences ranging in length from 140 to 185 aa within only a few minutes. The 3D protein structures of these sequences were predicted by ColabFold, which offered fast structure prediction based on AlphaFold2 and an orthogonal validation of the putative folding of sequences generated by the RF_joint_. The quality of the prediction was evaluated by the per‐residue confidence score predicted LDDT (pLDDT) (estimating how well the prediction would agree with an experimental structure) and template modeling scores (measuring the similarity of the predicted structure to a known template). The predictions with a pLDDT > 70 and a pTM score > 0.7 were kept for further screening. In order to further narrow down the candidate pool of the virtual enzymes that are potentially active, we screened them according to the accuracy of motif recapitulation, the interactions between the model substrate 2‐HE(MHET)_3_ and the virtual enzymes, and the structural stability in MD simulations. The accuracy of motif recapitulation was assessed by the motif RMSD between the original motifs from LCC and those from the virtual enzymes. The virtual enzymes with motif RMSD larger than 2.5 Å were discarded.^[^ ^29^ ^]^ **Figure** **2**a shows a 3D scatter plot of 75 designs, of which 44 meet these static structural quality criteria (blue dots). The right upper panel depicts a typical structural superposition of the retained segments in a predicted protein structure against the template, where the motif RMSD is only 2.0 Å, indicating a successful motif recapitulation. The filtered virtual enzymes were further examined via molecular docking with 2‐HE(MHET)_3_. A typical docking result is shown in Figure 2b. As the binding energy between LCC and 2‐HE(MHET)_3_ was calculated to be −2.78 kcal mol^−1^, we chose a range of −4–0 kcal mol^−1^ as a screening criterion. Furthermore, the reaction mechanism necessitates two criteria: the distance between the carbonyl carbon atom of 2‐HE(MHET)_3_ and the oxygen atom of serine (Ser) (< 4 Å), and the potential to form an oxyanion hole by the backbone NH groups.^[^ ^30^ ^]^ The structural stability of the virtual enzymes was assessed by calculating the Cα‐RMSD evolution through MD simulations. The virtual enzymes whose RMSD values exceed 5 Å within a 20 ns simulation were discarded (Figure 2c). Ten virtual enzymes (designated as P1 through P10) out of 100 candidates passed the computational screening steps described above. The molecular docking of these enzymes with 2‐HE(MHET)_3_ is shown in Figure S1 (Supporting Information). All these enzymes are 28–46% shorter in length than LCC, with full lengths between 140 and 186 and molecular weights between 14.28 and 19.31 kDa. Detailed descriptions including their sequences, lengths, and molecular weights are summarized in Table S1 (Supporting Information), and the predicted protein structures are shown in Figure S2 (Supporting Information).

### 2.3 Expression and Experimental Characterization of the First‐Round Designed PET Hydrolases

The ten virtual enzymes were expressed in the *E. coli* BL21 (DE3) strain. The enzymes, P1 to P10, were fused with His‐tags at the N‐termini, allowing facile purification by Ni‐NTA chromatography (Figure 2d). SDS‐PAGE analysis revealed that only P3 formed inclusion bodies, whereas no inclusion bodies were observed in the cell lysates of the other proteins (Figure S3a,b, Supporting Information). All eluting protein fractions contained only a small fraction of the target enzyme, along with a substantial amount of protein impurities, indicating that the target proteins were poorly expressed (less than 100 ng mL^−1^ in the cell lysates except for P3). The low expression levels imply a low quality of the in silico designed sequences. Nevertheless, we tested the activity of these enzymes in the protein mixture. P1, P3, P4, P5, P6, P7, P8, and P9 were found to be active for hydrolyzing bis(2‐hydroxyethyl) terephthalate (BHET) into mono(2‐hydroxyethyl) terephthalate (MHET) and terephthalate (TPA), suggesting that these designer enzymes are esterases (Figure 2e). To test if these enzymes have PET hydrolytic activity, we prepared a suspension of PET‐NPs as the substrate.^[^ ^31^ ^]^ The as‐prepared PET‐NPs are nanospheres with an average size of 145 ± 50 nm (Figure 2f,g). We found that P1, P3, P4, P5, P6, P7, P8, and P10 could hydrolyze PET‐NPs into MHET and TPA, as characterized by high‐performance liquid chromatography (HPLC) (Figure 2h). The success in reproducing the hydrolytic activity for PET‐NPs indicates that our design workflow encapsulates some essence of PET hydrolases. However, none of these enzymes exhibited hydrolytic activity against micronized PET microparticles (≈50 µm in size and 19% crystallinity), as shown in Figure S4 (Supporting Information).

### 2.4 Rescuing the Designs by Iteratively Running RF_joint_

**Figure 3:**
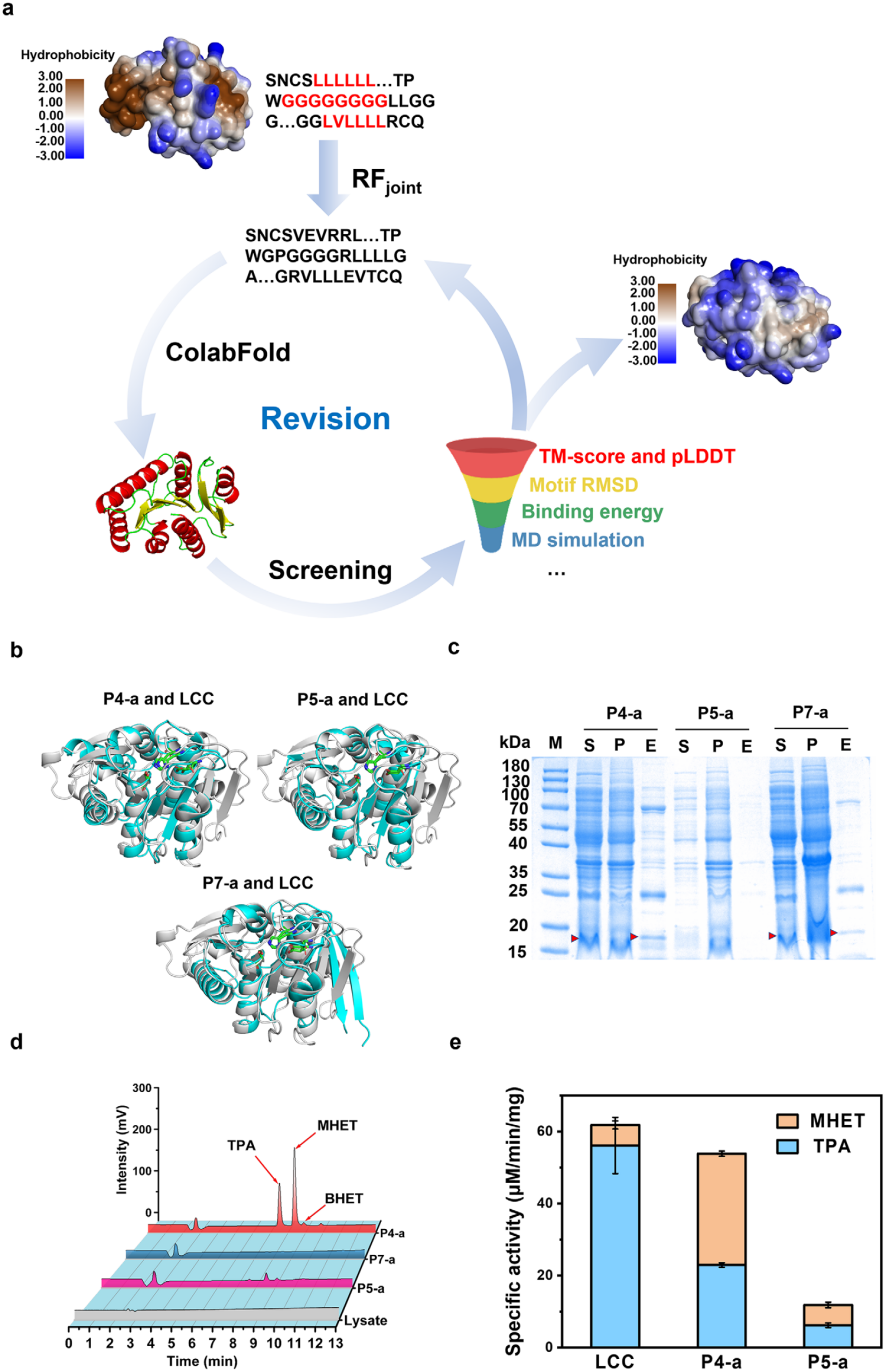
Computational refinement of the designed sequences. a) The exposed hydrophobic regions (brown) on the protein surface of and the amino acid sequences of SAAR and CHAA patterns (red) were redesigned by RF_joint_, followed by 3D structure prediction and computational screening. The example shown in the left panel is P7. b) The structural comparisons between P4‐a, P5‐a, P7‐a (white) and the template enzyme LCC (blue), respectively. c) SDS‐PAGE analysis of P4‐a, P5‐a and P7‐a, where S represents the soluble fraction of the cell lysate, P represents the precipitates of the cell lysate, E represents the eluted fraction from the Ni‐NTA column. d) Hydrolysis of PET microparticles by P4‐a, P5‐a and P7‐a at 60 °C for 24 h. The hydrolysis by the cell lysate of *E. coli* carrying empty plasmids is provided as a control. e) Specific activities of LCC, P4‐a and P5‐a against PET microparticles.

We assumed that the poor expressibility and the absence of hydrolytic activity on PET microparticles were due to the inherent flaws in the sequences designed in the first round. A review of the sequences revealed that the RF_joint_ algorithm tends to produce long single amino acid repeats (SAAR) such as LLLLLLL and GGGGGGGG, and consecutive hydrophobic amino acids (CHAA) such as LVVLV, which are uncommon in native protein sequences (Table S2, Supporting Information). The lack of amino acid diversity in the generated sequences has been discussed by Zheng et al. as an inherent limitation of structure‐based protein sequence design models.^[^ ^32^ ^]^ These unusual patterns may significantly influence the foldability of our designs, especially given their relatively small sizes. In the predicted 3D structures of virtual proteins, we found large regions of hydrophobic patches on the protein surface, which are most likely the source of protein misfolding and aggregation.^[^ ^33^ ^]^ These regions may be identified as aberrant by the cellular proteolytic machineries, such as the Lon protease, leading to a rapid degradation of the as‐synthesized peptides.^[^ ^34^ ^]^ Therefore, we iteratively employ RF_joint_ to generate new sequences to replace the problematic ones encoding long SAAR and CHAA patterns and large hydrophobic patches (**Figure** **3**a). Typically, several runs of iterations were enough to replace the problematic areas to the maximum degree that RF_joint_ can repair. The redesigned sequences were filtered by the aforementioned computational screening criteria. We obtained three new designer enzymes, namely P4‐a (derived from P4), P5‐a (derived from P5), and P7‐a (derived from P7), as shown in Figure S5 (Supporting Information). The surface hydrophobic area of the designer enzymes (Table S3, Supporting Information) significantly decreases after sequence refinement, making the designs more akin to natural proteins. The regional pLDDT values of the redesigned sequences as well as the overall pLDDT and pTM‐scores of the full‐length sequences are significantly improved (Figure S6 and Table S1, Supporting Information), indicating a higher structural quality predicted by ColabFold. The theoretical molecular weights of these enzymes were 19.43, 18.47, and 18.34 kDa, respectively, which are 30–34% smaller than the molecular weight of LCC. The three designer enzymes share only 47%, 46%, and 47% sequence similarities compared to LCC (Figure 3b; Figure S7, Supporting Information). Nevertheless, the molecular docking of these enzymes with 2‐HE(MHET)_3_ show that the substrate molecule is accommodated in the active site with appropriate interactions for catalysis (Figure S8, Supporting Information).

We then expressed P4‐a, P5‐a, and P7‐a in the Lemo21 (DE3) competent *E. coli* strain, a BL21 (DE3) derivative harboring the pLemo plasmid, which is suitable for the expression of toxic proteins, membrane proteins and proteins with solubility problems.^[^ ^35^ ^]^ After the expression and purification, the eluting proteins were subjected to SDS‐PAGE analysis (Figure 3c). Unfortunately, the expression of these enzymes was not improved substantially, and the band for P5‐a was almost invisible. While the BHET hydrolytic activity was verified for P4‐a and P7‐a, it was not observed for P5‐a because of its extremely low expression (Figure S9, Supporting Information). We determined the activity of these new designer enzymes (in the eluted fraction) using PET microparticles as the substrate and found that both P4‐a and P5‐a exhibit PET hydrolytic activity, while P7‐a shows no activity. Subsequently, we focused on enhancing the expressibility of P4‐a and P5‐a designs (Figure 3d).

The expression problem has occurred in other PET hydrolases, such as those mined from the human saliva metagenome.^[^ ^19^ ^]^ Fusion to SUMO (small ubiquitin‐like modifier) tags is considered effective in boosting the expression level of recombinant proteins by improving folding, solubility, and stability.^[^ ^36^ ^]^ We therefore expressed SUMO‐tagged P4‐a and SUMO‐tagged P5‐a in *E. coli* BL21 (DE3) PLysS cells (Figure S10, Supporting Information). The SUMO tags on P4‐a and P5‐a were removed after nickel‐nitrilotriacetic acid (Ni‐NTA) purification by adding SUMO‐specific proteases ULP1 (ubiquitin‐like‐specific protease 1). SUMO‐fusion increased the expression levels of both P4‐a and P5‐a by several folds, as demonstrated by the enhanced hydrolysis of the PET microparticles using the protein elution from the Ni‐NTA column (Figure S11, Supporting Information). Through quantitative western blotting, we quantified the concentrations of P4‐a and P5‐a (with SUMO tags) after the Ni‐NTA affinity purification to be 1.7 and 2.9 µm, respectively (Figure S10b,c, Supporting Information). After the cleavage of SUMO tags, the specific activity of P4‐a and P5‐a was determined with PET microparticles. As shown in Figure 3d, P4‐a exhibits higher PET hydrolytic activity than P5‐a. While its PET degradation activity is comparable to that of LCC, P4‐a demonstrates compromised activity toward MHET. The temperature optima of P4‐a and P5‐a are both 60 °C, demonstrating that these two designer enzymes have excellent thermostability (Figure S12, Supporting Information).

### 2.5 Rescuing the Designs using ProteinMPNN Algorithm

**Figure 4:**
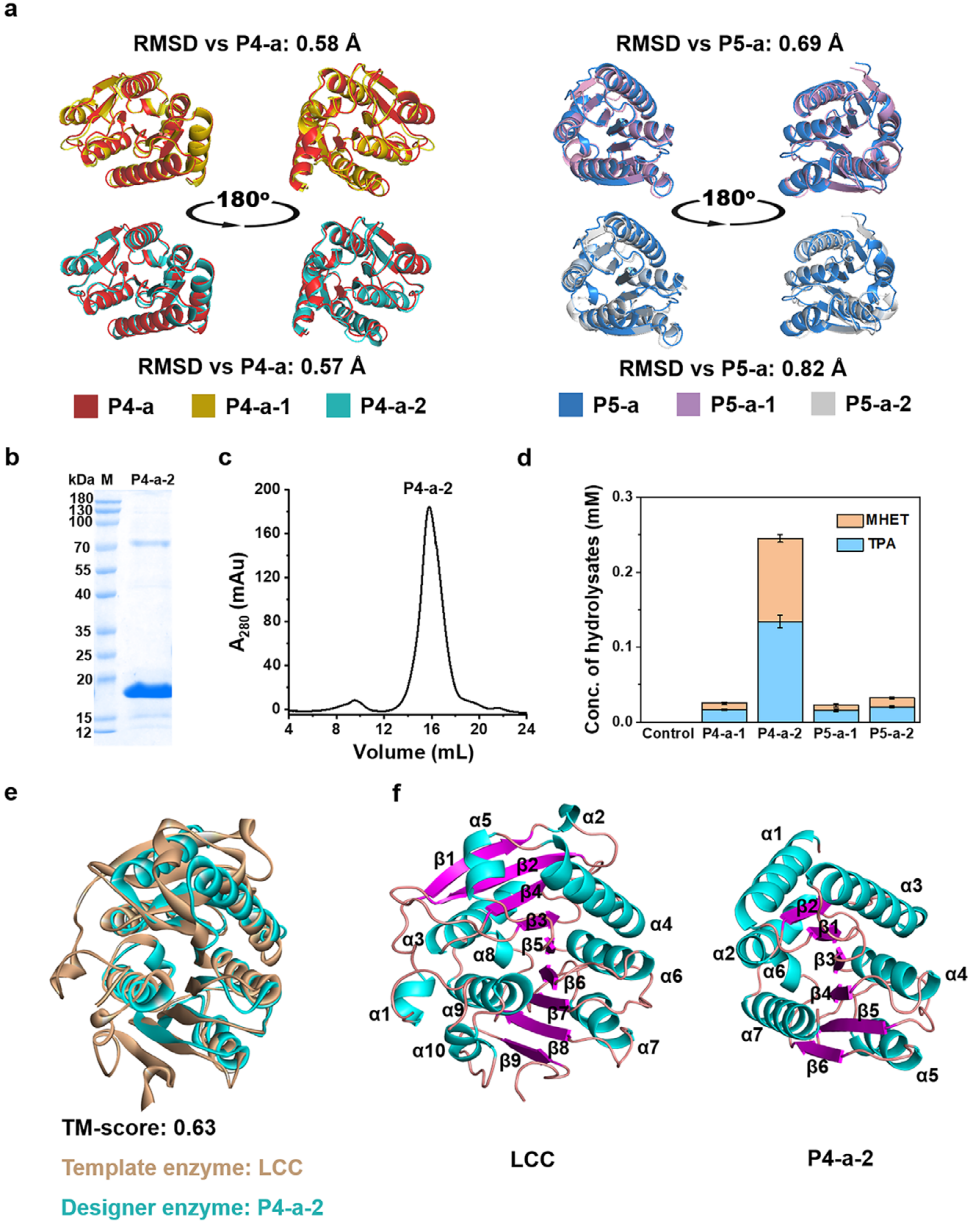
Improving protein expression by ProteinMPNN redesign. a) Superimposition of the 3D structures of redesigned proteins on the corresponding original protein. P4‐a‐1 and P4‐a‐2 (also named *Rs*PETase1) are redesigned from P4‐a; P5‐a‐1 and P5‐a‐2 are redesigned from P5‐a. Protein structures were predicted by ColabFold. b) Coomassie‐stained SDS‐PAGE analysis of *Rs*PETase1. c) Size‐exclusion chromatography of *Rs*PETase1. d) Concentrations of hydrolysates released from the hydrolysis of PET microparticles by the redesigned enzymes. The hydrolysis was carried out with 6.0 µg mL^−1^ eluted protein fractions from a Ni‐NTA affinity column and 5.0 mg mL^−1^ PET microparticles at 50 °C for 24 h. The lysate of *E. coli* cells carrying empty plasmids was used as the control. e) Structural alignment of LCC and *Rs*PETase1. f) Ribbon diagrams of LCC (left) and *Rs*PETase1 (right) with β‐strands labeled in magenta and α‐helices in cyan.

The expression levels of SUMO‐tagged proteins were still insufficient for further purification and practical application. The difficulty in expression of the above enzymes indicates the presence of elusive imperfections of the computationally generated sequences, which is a common issue of *de novo* designed proteins.^[^ ^37^ ^]^ Dauparas et al. developed ProteinMPNN to rescue failed designs by Rosetta or AlphaFold, enhancing their solubility and thermostability.^[^ ^25^, ^38^ ^]^ Thus, we employed ProteinMPNN to optimize the sequences of the potentially active designer enzymes P4‐a and P5‐a based on their respective backbones. In this process, we did not fix the catalytic center but performed the global sequence optimization using ProteinMPNN. We generated ten new sequences for each backbone and predicted their 3D structures using ColabFold. After in silico screening via the above‐mentioned computational approaches, we finally selected four sequences for expression in the *E. coli* BL21 (DE3) strain, namely P4‐a‐1 and P4‐a‐2 (derived from P4‐a), P5‐a‐1, and P5‐a‐2 (derived from P5‐a). The backbone RMSD values of these proteins before and after optimization by ProteinMPNN are less than 1.0 Å (**Figure** **4**a), and the optimized structures exhibit increased structural quality and stability (Figures S13 and S14, Supporting Information). The expression level of P4‐a‐2 was significantly improved to ≈2.5 mg L^−1^, yielding 95% purity after Ni‐NTA chromatography (Figure 4b,c). The other three proteins, however, were almost invisible in the SDS‐PAGE analysis (Figure S15a,b, Supporting Information). All redesigned enzymes (tested as crude cell lysates) exhibited hydrolytic activities toward BHET (Figure S16, Supporting Information) and PET microparticles (Figure 4d).

We evaluated the sequence and structural similarities of P4‐a‐2 with the template enzyme LCC. Pairwise sequence alignment shows that P4‐a‐2 shares only 34% sequence similarity with LCC and 25% with *Is*PETase, respectively (Figure S17, Supporting Information). This implies that the catalytic function can be maintained even if a significant portion of protein sequences is altered. From a structural perspective, P4‐a‐2 still belongs to the α/β hydrolase superfamily, sharing a common fold with other hydrolases that typically consist of a core of β‐sheets packed between two layers of α‐helices. The structural similarity is assessed through the structural alignment of P4‐a‐2 and LCC using TM‐align,^[^ ^39^ ^]^ producing a TM‐score of 0.63 (Figure 4e). LCC features nine β‐strands sandwiched by ten α‐helices, with the core being structurally conserved and identical to *Is*PETase.^[^ ^40^ ^]^ In comparison, P4‐a‐2 is a more compact enzyme composed of a central β‐sheet with six β‐strands flanked by seven α‐helices (Figure 4f; Figure S18, Supporting Information). Given the sequence and structural differences described above, we consider P4‐a‐2 to be a new‐to‐nature PET hydrolase. This indicates that the inpainting algorithm has learned essential features of α/β hydrolase fold, although it generates sequences rather than structures. The recapitulation of functional motifs by our workflow does not merely copy the sequences or structures of α/β‐hydrolases, but somehow reflects the underlying sequence‐structure‐function relationship. As P4‐a‐2 is the first designer PETase created by computational *Re‐scaffolding*, we refer to it as *Rs*PETase1.

**Figure 5:**
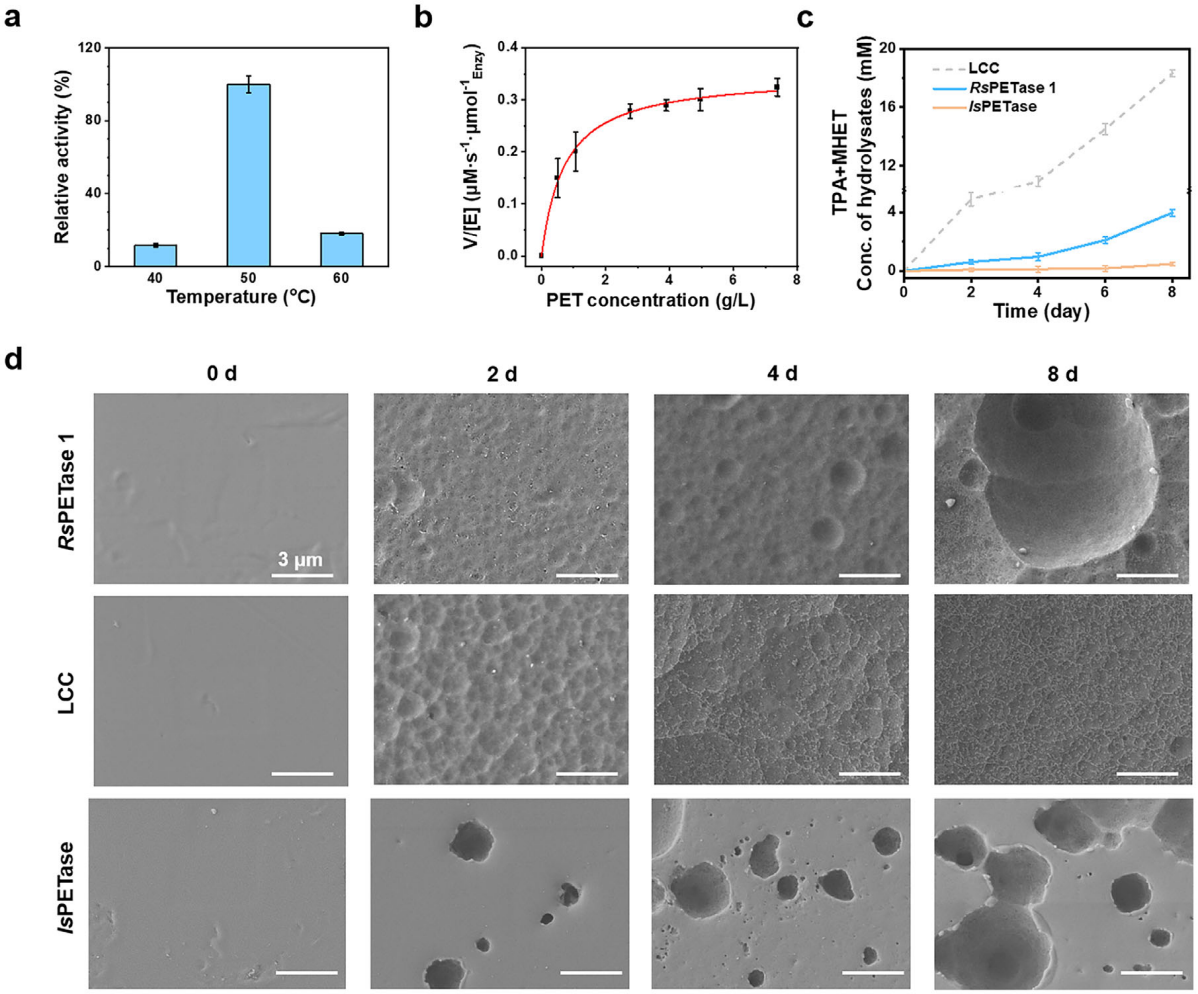
PET hydrolytic performances of *Rs*PETase1. a) Temperature dependence of *Rs*PETase1 for the hydrolysis of PET microparticles. The hydrolysis was carried out with 6.0 µg mL^−1^ enzyme and 5 mg mL^−1^ PET microparticles at varied temperatures and the activity was determined based on the released amount of hydrolysates in 24 h. b) Michaelis–Menten plot of *Rs*PETase1 assayed at 50 °C. c) Time courses of PET film hydrolysis into soluble hydrolysates MHET and TPA by *Rs*PETase1, LCC or *Is*PETase within 8 days at 50 °C. The PET film was cut into disks with a diameter of 6 mm and in 50 mm phosphate buffer at a concentration of 5 mg mL^−1^. Enzymes were refreshed every 48 h. d) SEM images of amorphous PET film from Goodfellow treated with *Rs*PETase1, LCC or *Is*PETase at an enzyme concentration of 90 µg mL^−1^ and a reaction temperature of 50 °C. Scale bars are 5 µm.

An excellent thermostaility is neccessary for PET hydrolases as they are supposed to hydrolyze PET at temperatures above the glass transition temperaure of PET.^[^ ^41^, ^42^ ^]^ Differential scanning fluorimetry (DSF) suggests that the *Rs*PETase1 has a melting temperature of 56 °C (Figure S19, Supporting Information), which is in good agreement with its optimal temperature of 50 °C for PET hydrolysis (**Figure** **5**a). Using the PET microparticles as the substrate, we analyzed the kinetic parameters of *Rs*PETase1 following the conventional Michaelis‐Menten model. Our findings revealed a maximum reaction rate (V_max_) of 0.55 µm min^−1^ and a *K*_m_ value of 0.72 g L^−1^, resulting in a catalytic efficiency of 25.5 L g^−1^ min^−1^ (Figure 5b). In comparison, under the same conditions, LCC exhibited a *V*_max_ five times greater and a *K*_m_ 5.5 times higher than those of *Rs*PETase1, indicating its catalytic efficiency similar to that of *Rs*PETase1 (Figure S20 and Table S7, Supporting Information). We conducted a long‐term hydrolysis of 5 mg mL^−1^ Goodfellow PET film using *Rs*PETase1, LCC and *Is*PETase, respectively. Over an 8‐day degradation period, *Rs*PETase1 produced 70% fewer hydrolysates compared to LCC, while produced eightfold more hydrolysates than *Is*PETase (Figure 5c). The films treated with LCC and *Rs*PETase1 became opaque during the hydrolysis, while the film treated with *Is*PETase remained transparent. The changes in surface morphology of the PET film were observed using scanning electron microscopy (SEM), as shown in Figure 5d. *Rs*PETase1 and LCC both induced significant morphological alterations to the PET surface compared to the buffer control. The films were initially etched to create pores, though LCC degraded PET faster than *Rs*PETase1. Over time, the entire surface layer was gradually etched, resulting in a rough texture characterized by pits and dents. In contrast, *Is*PETase tended to enlarge the existing cavities rather than uniformly etch the surface. The surface erosion mechanism of *Rs*PETase1 more resembles that of LCC. Considering the remarkable achievements of recently reported mutants like HotPETase,^[^ ^42^ ^]^ FAST‐PETase^[^ ^43^ ^]^ that exhibit three orders of magnitude higher PET hydrolytic activity than their wild‐types,^[^ ^44^, ^45^ ^]^ we believe that the PET hydrolytic activity of *Rs*PETase1 can also be significantly enhanced through several rounds of protein engineering.

## 3 Conclusion

We have demonstrated a successful workflow for the computational design of new‐to‐nature PET hydrolases by scaffolding the known active sites using a combination of recently published deep learning algorithms. The design leverages RF_joint_ to generate diverse backbone structures that support the catalytic motifs of a known PET hydrolase, exploring a broad range of structural possibilities while preserving the active site essential for PET hydrolysis. A set of physiochemical criteria was established for computational screening of putatively foldable and active designer enzymes. The filtered designs were validated by expression in *E. coli*. cells. Failed designs were rescued by iterative inpainting using RF_joint_ and sequence redesign using ProteinMPNN, which can generate high‐quality sequences that can fold into the designed protein backbones.^[^ ^25^ ^]^ This synergistic approach enhances the experimental success rate of protein design by combining structural flexibility with foldability. We obtained three novel PET hydrolases, P4‐a, P5‐a, and P4‐a‐2, and were able to efficiently express P4‐a‐2 (also named as *Rs*PETase1). These designer enzymes show low sequence and structural similarities compared to currently known PET hydrolases, manifesting the ability of deep learning algorithms to construct new‐to‐nature enzymes without relying on the existing protein scaffolds evolved by nature. Notably, compared to the template enzyme LCC, *Rs*PETase1 exhibits comparable catalytic efficiency, although its *V*_max_ is compromised. Additionally, it has reasonably high thermostability, highlighting its strong potential for further engineering in industrial applications. This work paves an avenue to computationally build up the diversity of enzymes with known catalytic mechanisms, through which one can screen more robust and efficient enzymes (or proteins of interest) of industrial or pharmaceutical importance.

The successful replication of enzymatic activity by recapitulation of functional motifs with the same coordinates of native enzymes reaffirms that catalysis is accomplished by a small fraction of residues.^[^ ^46^, ^47^ ^]^ On the other hand, different protein scaffolds possess distinct inherent protein dynamics, including the atomic local fluctuations and large structure collective motions, which also significantly regulates the intrinsic activity of enzymes.^[^ ^48^ ^]^ The computational construction of isoenzymes with artificial protein architectures can yield isoenzymes with distinct protein dynamics that are absent in nature. With these isoenzymes we are able to pursue quantitative and mechanistic insights into the relationship between catalysis and protein dynamics.

The rapid development of the *de novo* design of proteins through deep learning models has also stoked the ambition to create small proteins or miniproteins with desired functions.^[^ ^16^, ^49^, ^50^ ^]^ Small proteins have considerable advantages over larger ones: their genes are easier to synthesize, they take up fewer cell resources to express, they can be engineered to be highly stable, and they are potentially able to penetrate into tissues and cells.^[^ ^51^, ^52^, ^53^ ^]^ In the context of catalysis, smaller enzymes duplicate the desired catalytic activity using just the necessary residues and may benefit the cost‐effectiveness of industrial applications. In this study, we intentionally generate new designs with shorter amino acid sequences compared to the original enzyme. The obtained active enzymes are 177 and 185 amino acids long, making them 30% smaller than the template enzyme LCC. The expression problems of some designs can be partly attributed to the smaller protein size, as the shortening of the polypeptide chains makes it more difficult to effectively bury hydrophobic amino acids in the protein core. The resulting exposure of hydrophobic areas increases the propensity for protein misfolding and aggregation. In addition, we found that the RF_joint_ model tends to “inpaint” missing regions with consecutively repeated and hydrophobic amino acids, making the design of small proteins even more challenging. ProteinMPNN is effective at enhancing protein solubility, though this often comes at the cost of reduced activity.^[^ ^38^ ^]^ The inherent limitations of current deep‐learning tools present challenges in protein design, especially for small enzymes. We anticipate that in the near future, the expansion of the small proteins database^[^ ^54^ ^]^ and the advancements of protein design models trained on such proteins will boost our ability to create artificial small proteins and enzymes with greater accuracy and success rate.

## Conflict of Interest

The authors declare no conflict of interest.

## Author Contributions

Y.Z. conceived and designed the research. Y.D. performed the computational design. Y.D. and S.Z. performed the experiments. Y.D and Y.Z. wrote the initial manuscript. Y.D., Y.Z., X.K., and H.H. discussed the results and revised the manuscript.

## Supporting information



Supporting Information

## Data Availability

The data that support the findings of this study are available from the corresponding author upon reasonable request.

## References

[1] C. Katsimpouras , G. Stephanopoulos , Curr. Opin. Biotechnol. 2021, 69, 91. 33422914 10.1016/j.copbio.2020.12.003

[2] E. L. Bell , W. Finnigan , S. P. France , A. P. Green , M. A. Hayes , L. J. Hepworth , S. L. Lovelock , H. Niikura , S. Osuna , E. Romero , K. S. Ryan , N. J. Turner , S. L. Flitsch , Nat. Rev. Methods Primers 2021, 1, 46.

[3] R. Buller , S. Lutz , R. J. Kazlauskas , R. Snajdrova , J. C. Moore , U. T. Bornscheuer , Science 2023, 382, eadh8615. 37995253 10.1126/science.adh8615

[4] I. G. Riziotis , A. J. M. Ribeiro , N. Borkakoti , J. M. Thornton , J. Mol. Biol. 2022, 434, 167517. 35240125 10.1016/j.jmb.2022.167517 PMC9005782

[5] P. F. Gherardini , M. N. Wass , M. Helmer‐Citterich , M. J. Sternberg , J. Mol. Biol. 2007, 372, 817. 17681532 10.1016/j.jmb.2007.06.017

[6] L. Jiang , E. A. Althoff , F. R. Clemente , L. Doyle , D. Röthlisberger , A. Zanghellini , J. L. Gallaher , J. L. Betker , F. Tanaka , C. F. Barbas 3rd , D. Hilvert , K. N. Houk , B. L. Stoddard , D. Baker , Science 2008, 319, 1387. 18323453 10.1126/science.1152692 PMC3431203

[7] D. Rothlisberger , O. Khersonsky , A. M. Wollacott , L. Jiang , J. DeChancie , J. Betker , J. L. Gallaher , E. A. Althoff , A. Zanghellini , O. Dym , S. Albeck , K. N. Houk , D. S. Tawfik , D. Baker , Nature 2008, 453, 190. 18354394 10.1038/nature06879

[8] A. Robles‐Martín , R. Amigot‐Sánchez , L. Fernandez‐Lopez , J. L. Gonzalez‐Alfonso , S. Roda , V. Alcolea‐Rodriguez , D. Heras‐Márquez , D. Almendral , C. Coscolín , F. J. Plou , R. Portela , M. A. Bañares , Á. Martínez‐del‐Pozo , S. García‐Linares , M. Ferrer , V. Guallar , Nat. Catal. 2023, 6, 1174.

[9] I. Kalvet , M. Ortmayer , J. Zhao , R. Crawshaw , N. M. Ennist , C. Levy , A. Roy , A. P. Green , D. Baker , J. Am. Chem. Soc. 2023, 145, 14307. 37341421 10.1021/jacs.3c02742 PMC10326885

[10] P. A. Alexander , Y. He , Y. Chen , J. Orban , P. N. Bryan , Proc. Natl. Acad. Sci., USA 2009, 106, 21149. 19923431 10.1073/pnas.0906408106 PMC2779201

[11] X. Pan , T. Kortemme , J. Biol. Chem. 2021, 296, 100558. 33744284 10.1016/j.jbc.2021.100558 PMC8065224

[12] A. W. Senior , R. Evans , J. Jumper , J. Kirkpatrick , L. Sifre , T. Green , C. Qin , A. Zidek , A. W. R. Nelson , A. Bridgland , H. Penedones , S. Petersen , K. Simonyan , S. Crossan , P. Kohli , D. T. Jones , D. Silver , K. Kavukcuoglu , D. Hassabis , Nature 2020, 577, 706. 31942072 10.1038/s41586-019-1923-7

[13] J. Jumper , R. Evans , A. Pritzel , T. Green , M. Figurnov , O. Ronneberger , K. Tunyasuvunakool , R. Bates , A. Zidek , A. Potapenko , A. Bridgland , C. Meyer , S. A. A. Kohl , A. J. Ballard , A. Cowie , B. Romera‐Paredes , S. Nikolov , R. Jain , J. Adler , T. Back , S. Petersen , D. Reiman , E. Clancy , M. Zielinski , M. Steinegger , M. Pacholska , T. Berghammer , S. Bodenstein , D. Silver , O. Vinyals , et al., Nature 2021, 596, 583. 34265844 10.1038/s41586-021-03819-2 PMC8371605

[14] J. Abramson , J. Adler , J. Dunger , R. Evans , T. Green , A. Pritzel , O. Ronneberger , L. Willmore , A. J. Ballard , J. Bambrick , S. W. Bodenstein , D. A. Evans , C. C. Hung , M. O'Neill , D. Reiman , K. Tunyasuvunakool , Z. Wu , A. Žemgulytė , E. Arvaniti , C. Beattie , O. Bertolli , A. Bridgland , A. Cherepanov , M. Congreve , A. I. Cowen‐Rivers , A. Cowie , M. Figurnov , F. B. Fuchs , H. Gladman , R. Jain , et al., Nature 2024, 630, 493. 38718835 10.1038/s41586-024-07487-w PMC11168924

[15] J. Wang , S. Lisanza , D. Juergens , D. Tischer , J. L. Watson , K. M. Castro , R. Ragotte , A. Saragovi , L. F. Milles , M. Baek , I. Anishchenko , W. Yang , D. R. Hicks , M. Exposit , T. Schlichthaerle , J. H. Chun , J. Dauparas , N. Bennett , B. I. M. Wicky , A. Muenks , F. DiMaio , B. Correia , S. Ovchinnikov , D. Baker , Science 2022, 377, 387. 35862514 10.1126/science.abn2100 PMC9621694

[16] A. H. Yeh , C. Norn , Y. Kipnis , D. Tischer , S. J. Pellock , D. Evans , P. Ma , G. R. Lee , J. Z. Zhang , I. Anishchenko , B. Coventry , L. Cao , J. Dauparas , S. Halabiya , M. DeWitt , L. Carter , K. N. Houk , D. Baker , Nature 2023, 614, 774. 36813896 10.1038/s41586-023-05696-3 PMC9946828

[17] A. Madani , B. McCann , N. Naik , N. S. Keskar , N. Anand , R. R. Eguchi , P.‐S. Huang , R. Socher , bioRxiv 2020, 03, 982272.

[18] A. L. Hansen , F. F. Theisen , R. Crehuet , E. Marcos , N. Aghajari , M. Willemoes , ACS Synth. Biol. 2024, 13, 862. 38357862 10.1021/acssynbio.3c00674 PMC10949244

[19] B. Eiamthong , P. Meesawat , T. Wongsatit , J. Jitdee , R. Sangsri , M. Patchsung , K. Aphicho , S. Suraritdechachai , N. Huguenin‐Dezot , S. Tang , W. Suginta , B. Paosawatyanyong , M. M. Babu , J. W. Chin , D. Pakotiprapha , W. Bhanthumnavin , C. Uttamapinant , Angew Chem., Int. Ed. 2022, 61, e202203061. 10.1002/anie.202203061 PMC7613822 35656865

[20] S. Yoshida , K. Hiraga , T. Takehana , I. Taniguchi , H. Yamaji , Y. Maeda , K. Toyohara , K. Miyamoto , Y. Kimura , K. Oda , Science 2016, 351, 1196. 26965627 10.1126/science.aad6359

[21] V. Tournier , S. Duquesne , F. Guillamot , H. Cramail , D. Taton , A. Marty , I. Andre , Chem. Rev. 2023, 123, 5612. 36916764 10.1021/acs.chemrev.2c00644

[22] S. Joo , I. J. Cho , H. Seo , H. F. Son , H. Y. Sagong , T. J. Shin , S. Y. Choi , S. Y. Lee , K. J. Kim , Nat. Commun. 2018, 9, 382. 29374183 10.1038/s41467-018-02881-1 PMC5785972

[23] S. Sulaiman , S. Yamato , E. Kanaya , J. J. Kim , Y. Koga , K. Takano , S. Kanaya , Appl. Environ. Microbiol. 2012, 78, 1556. 22194294 10.1128/AEM.06725-11 PMC3294458

[24] M. Mirdita , K. Schutze , Y. Moriwaki , L. Heo , S. Ovchinnikov , M. Steinegger , Nat. Methods. 2022, 19, 679. 35637307 10.1038/s41592-022-01488-1 PMC9184281

[25] J. Dauparas , I. Anishchenko , N. Bennett , H. Bai , R. J. Ragotte , L. F. Milles , B. I. M. Wicky , A. Courbet , R. J. de Haas , N. Bethel , P. J. Y. Leung , T. F. Huddy , S. Pellock , D. Tischer , F. Chan , B. Koepnick , H. Nguyen , A. Kang , B. Sankaran , A. K. Bera , N. P. King , D. Baker , Science 2022, 378, 49. 36108050 10.1126/science.add2187 PMC9997061

[26] S. Sulaiman , D. J. You , E. Kanaya , Y. Koga , S. Kanaya , Biochemistry 2014, 53, 1858. 24593046 10.1021/bi401561p

[27] X. Han , W. Liu , J. W. Huang , J. Ma , Y. Zheng , T. P. Ko , L. Xu , Y. S. Cheng , C. C. Chen , R. T. Guo , Nat. Commun. 2017, 8, 2106. 29235460 10.1038/s41467-017-02255-z PMC5727383

[28] V. Tournier , C. M. Topham , A. Gilles , B. David , C. Folgoas , E. Moya‐Leclair , E. Kamionka , M. L. Desrousseaux , H. Texier , S. Gavalda , M. Cot , E. Guemard , M. Dalibey , J. Nomme , G. Cioci , S. Barbe , M. Chateau , I. Andre , S. Duquesne , A. Marty , Nature 2020, 580, 216. 32269349 10.1038/s41586-020-2149-4

[29] I. G. Riziotis , A. J. M. Ribeiro , N. Borkakoti , J. M. Thornton , J. Mol. Biol. 2023, 435, 168254. 37652131 10.1016/j.jmb.2023.168254

[30] C. Jerves , R. P. P. Neves , M. J. Ramos , S. da Silva , P. A. Fernandes , ACS. Catal. 2021, 11, 11626.

[31] L. M. Johnson , J. B. Mecham , S. A. Krovi , M. M. Moreno Caffaro , S. Aravamudhan , A. L. Kovach , T. R. Fennell , N. P. Mortensen , Nanoscale. Adv. 2021, 3, 339. 36131728 10.1039/d0na00888e PMC9417664

[32] Z. X. Zheng , Y. F. Deng , D. Y. Xue , Y. Zhou , F. Ye , Q. Q. Gu , bioRxiv 2023, 02, 526917.

[33] A. Kumar , N. K. Singh , D. Ghosh , M. Radhakrishna , Phys. Chem. Chem. Phys. 2021, 23, 12620. 34075973 10.1039/d1cp00954k

[34] S. A. Mahmoud , P. Chien , Annu. Rev. Biochem. 2018, 87, 677. 29648875 10.1146/annurev-biochem-062917-012848 PMC6013389

[35] S. Schlegel , J. Lofblom , C. Lee , A. Hjelm , M. Klepsch , M. Strous , D. Drew , D. J. Slotboom , J. W. de Gier , J. Mol. Biol. 2012, 423, 648. 22858868 10.1016/j.jmb.2012.07.019

[36] T. R. Butt , S. C. Edavettal , J. P. Hall , M. R. Mattern , Protein. Expr. Purif. 2005, 43, 1. 16084395 10.1016/j.pep.2005.03.016 PMC7129290

[37] D. Baker , Protein. Sci. 2019, 28, 678. 30746840 10.1002/pro.3588 PMC6423711

[38] K. H. Sumida , R. Nunez‐Franco , I. Kalvet , S. J. Pellock , B. I. M. Wicky , L. F. Milles , J. Dauparas , J. Wang , Y. Kipnis , N. Jameson , A. Kang , J. De La Cruz , B. Sankaran , A. K. Bera , G. Jimenez‐Oses , D. Baker , J. Am. Chem. Soc. 2024, 146, 2054. 38194293 10.1021/jacs.3c10941 PMC10811672

[39] Y. Zhang , Nucleic Acids Res. 2005, 33, 2302. 15849316 10.1093/nar/gki524 PMC1084323

[40] K. Hasan , M. Ulfah , N. Nurhayati , G. C. Sabbathini , S. R. Wulandari , I. G. E. P. Putra , I. Helianti , HAYATI J. Biosci. 2023, 31, 348.

[41] H. Hong , D. Ki , H. Seo , J. Park , J. Jang , K.‐J. Kim , Nat. Commun. 2023, 14, 4556. 37507390 10.1038/s41467-023-40233-w PMC10382486

[42] E. L. Bell , R. Smithson , S. Kilbride , J. Foster , F. J. Hardy , S. Ramachandran , A. A. Tedstone , S. J. Haigh , A. A. Garforth , P. J. R. Day , C. Levy , M. P. Shaver , A. P. Green , Nat. Catal. 2022, 5, 673.

[43] H. Lu , D. J. Diaz , N. J. Czarnecki , C. Zhu , W. Kim , R. Shroff , D. J. Acosta , B. R. Alexander , H. O. Cole , Y. Zhang , N. A. Lynd , A. D. Ellington , H. S. Alper , Nature 2022, 604, 662. 35478237 10.1038/s41586-022-04599-z

[44] G. Arnal , J. Anglade , S. Gavalda , V. Tournier , N. Chabot , U. T. Bornscheuer , G. Weber , A. Marty , ACS. Catal. 2023, 13, 13156. 37881793 10.1021/acscatal.3c02922 PMC10594578

[45] S. Kaabel , J. P. D. Therien , C. E. Deschenes , D. Duncan , T. Friscic , K. Auclair , Proc. Natl. Acad. Sci., USA 2021, 118, e2026452118. 34257154 10.1073/pnas.2026452118 PMC8307448

[46] A. J. M. Ribeiro , J. D. Tyzack , N. Borkakoti , G. L. Holliday , J. M. Thornton , J. Biol. Chem. 2020, 295, 314. 31796628 10.1074/jbc.REV119.006289 PMC6956550

[47] G. J. Bartlett , C. T. Porter , N. Borkakoti , J. M. Thornton , J. Mol. Biol. 2002, 324, 105. 12421562 10.1016/s0022-2836(02)01036-7

[48] K. Nam , M. Wolf‐Watz , Struct. Dyn. 2023, 10, 014301. 36865927 10.1063/4.0000179 PMC9974214

[49] K. Ozga , L. Berlicki , ACS Bio. Med. Chem. Au. 2022, 2, 316. 10.1021/acsbiomedchemau.2c00008 PMC10125317 37102166

[50] D. E. Kim , D. R. Jensen , D. Feldman , D. Tischer , A. Saleem , C. M. Chow , X. Li , L. Carter , L. Milles , H. Nguyen , A. Kang , A. K. Bera , F. C. Peterson , B. F. Volkman , S. Ovchinnikov , D. Baker , Proc. Natl. Acad. Sci., USA 2023, 120, e2207974120. 36897987 10.1073/pnas.2207974120 PMC10089152

[51] M. Jiang , H. Lou , W. Hou , Genome Instability & Disease 2021, 2, 225.

[52] Z. R. Crook , N. W. Nairn , J. M. Olson , Trends. Biochem. Sci. 2020, 45, 332. 32014389 10.1016/j.tibs.2019.12.008 PMC7197703

[53] L. Cao , I. Goreshnik , B. Coventry , J. B. Case , L. Miller , L. Kozodoy , R. E. Chen , L. Carter , A. C. Walls , Y. J. Park , E. M. Strauch , L. Stewart , M. S. Diamond , D. Veesler , D. Baker , Science 2020, 370, 426. 32907861 10.1126/science.abd9909 PMC7857403

[54] Y. Li , H. Zhou , X. Chen , Y. Zheng , Q. Kang , D. Hao , L. Zhang , T. Song , H. Luo , Y. Hao , R. Chen , P. Zhang , S. He , Genomics Proteomics Bioinform. 2021, 19, 602. 10.1016/j.gpb.2021.09.002 PMC9039559 34536568

