# Supplementary material for: Replicating PET Hydrolytic Activity by Positioning Active Sites with Smaller Synthetic Protein Scaffolds
Source: Adv Sci (Weinh). 2025 Mar 16;12(18):2500859. doi: 10.1002/advs.202500859 (PMC12079439; doi:10.1002/advs.202500859)
Supplement: Supplementary file 1 — Supporting Information [file ADVS-12-2500859-s001.pdf]

ADVANCED SCIENCE
Open Access

Supporting Information

for *Adv. Sci.*, DOI 10.1002/advs.202500859

Replicating PET Hydrolytic Activity by Positioning Active Sites with Smaller Synthetic Protein Scaffolds

*Yujing Ding, Shanshan Zhang, Xian Kong, Henry Hess and Yifei Zhang**

Supporting Information

Replicating PET hydrolytic activity by positioning active sites with de novo designed protein scaffolds

Yujing Ding[1,2], Shanshan Zhang[1,2], Xian Kong[3], Henry Hess[4], Yifei Zhang[1,2,*]

1. *State Key Laboratory of Chemical Resources Engineering, Beijing University of Chemical Technology, Beijing 100029, China*
2. *Beijing Advanced Innovation Center for Soft Matter Science and Engineering, Beijing University of Chemical Technology, Beijing 100029, China*
3. *South China Advanced Institute for Soft Matter Science and Technology, Guangdong Provincial Key Laboratory of Functional and Intelligent Hybrid Materials and Devices, School of Emergent Soft Matter, South China University of Technology, Guangzhou 510640, P. R. China*
4. *Department of Biomedical Engineering, Columbia University, 351L Engineering Terrace, 1210 Amsterdam Avenue, New York, NY 10027, United States.*

* E-mail: yifeizhang@mail.buct.edu.cn

Materials. Bis(2-Hydroxyethyl) terephthalate (BHET) was purchased from Shanghai Macklin Biochemical Technology Co., Ltd. The PET film was purchased from Goodfellow Co., Ltd. Kanamycin and β-D-1-thiogalactopyranoside (IPTG) were purchased from Yuanye Bio-Technology Co., Ltd. (Shanghai, China). Luria–Bertani (LB) was purchased from Sangon Biotech Co., Ltd. (Shanghai, China). All chemicals and reagents are of analytical grade.

All DNA primers and gene synthesis were completed by the Beijing Genomics Institute. DNA modifying enzymes and T4 DNA ligase were purchased from New England Biolabs. Plasmid extraction and gel purification kits were bought from Omega. Lemo21 (DE3) competent *E. coli* cells were purchased from Beijing Zoman Biotechnology Co., Ltd. The *E. coli* BL21 (DE3), *E. coli* BL21 (DE3) PLysS cells, *E. coli* Trans5α cells, plasmid pET28a (+) and DNA polymerase (2× PrimeSTAR Max Premix) were purchased from Tsingke Biology Co., Ltd. Cells were cultured in Luria–Bertani (LB) medium.

Methods

Plasmid construction and enzyme expression. All designs tested in *E. coli* were cloned, expressed and purified using standard methods. All genes encoding the designer proteins were synthesized by Beijing Genomics Institute. Genes encoding the first-round designed proteins (P1–P10) and the refined proteins (P4-a, P5-a and P7-a) were inserted into modified pET28a (+) vectors containing a N-terminal His_6-tags. Genes encoding proteins P4-a and P5-a were also inserted into modified pET28a (+) vectors containing N-terminal SUMO cleavage sites and a His_6-tags. Genes encoding proteins P4-a-1, P4-a-2, P5-a-1, and P5-a-2 were inserted into pET29b (+) vectors. The constructed plasmids were transformed into corresponding *E. coli* strain (Table S5). After an overnight culture on a Luria-Bertani (LB) agar plate containing 50 μg/mL kanamycin, a single colony was selected and inoculated into 3 mL of LB medium with 50 μg/mL kanamycin and incubated overnight at 37 °C with shaking at 200 rpm. The cells were then cultivated in 300 mL LB medium containing 50 μg/mL kanamycin at 37 °C/200 rpm until an OD_{600} reached above 0.6–0.8. Enzymes expression was induced by 0.5 mM IPTG at 16 °C for 18 h. The bacterial cells were harvested by centrifugation at 4000 × g for 30 min at 4 °C and resuspended in a lysis buffer (50 mM Tris-HCl, 300 mM NaCl and 10 mM imidazole at pH 8.0). The resuspended cells are disrupted by a benchtop high pressure homogenizer at 4 °C. Cell debris was removed by centrifugation at 4 °C, 10000 × g for 30 min. The

supernatant was incubated with a Ni-NTA resin for 1 h at 4 °C. The non-specifically adsorbed proteins were removed by washing with 50 mL wash buffer (50 mM Tris-HCl, 300 mM NaCl and 50 mM imidazole at pH 8.0). Then, the His-tagged protein was eluted with elution buffer (50 mM Tris-HCl, 300 mM NaCl and 300 mM imidazole at pH 8.0). The purified proteins were concentrated using a 10 kDa Amicon Ultra centrifuge tube (Millipore, Burlington, USA) and was then exchanged with sodium phosphate buffer (50 mM, pH 8.0) by passing through the HiTrap desalting column. Before the enzyme reaction, the enzyme concentration was quantified by the BCA Protein Assay Kit.

Cloning. P4-a and P5-A fused to SUMO were cloned into pET28a/His-SUMO A vector. The genes gP4-a /gP5-a for P4-a and P5-a were amplified from plasmids synthesized by the Beijing Genomics Institute. All primer informations are listed in Table S6**.** To obtain linearized pET28a His-SUMO A vector, a 50 μL polymerase chain reaction (PCR) system was prepared containing 9.5 ng pET28a/His-SUMO A vector plasmid, 0.5 μM primers (for both forward and reverse primers) and 25 μL 2× High-Fidelity Master Mix. The PCR was carried out on a Thermo Scientific Arktik thermal cycler (98 °C for 2 min; 98 °C for 10 s, 55 °C for 5 s, 72 °C for 30 s, 33 cycles; 72 °C for 5 min; 4 °C hold). The PCR product was digested by *Bam*H I and *Xho* I (100 U, NEB) at 37 °C for 2 h to degrade the template vector, and was then purified using PCR Purification Kit.

The linearized pET28a/His-SUMO A vector and insert genes (gP4-a/gP5-a) were assembled at 22 °C for 1.5 h in a 10 μL reaction system, containing the vector (5 ng), inserts (1.5 ng gP4-a or 1.5 ng gP5-a) and 1 μL T4 DNA ligase with 1 μL 10× T4 DNA ligase buffer. The assembly products were transformed into 25 μL *E. coli* Trans5α Cells by electroporation using Eppendorf Eporator. The cells were then mixed with 400 μL LB medium and incubated at 37 °C with shaking for 50 min. The cells were cultured on LB agar plates with ampicillin (0.05 mg/mL), and the colonies were selected were inoculated in 5 mL LB broth with antibiotics and incubated at 37 °C with 200 rpm shaking overnight. The plasmid DNA was extracted using Plasmid Mini Kit. Colonies resulting from transformation of the ligation were screened by colony PCR, restriction enzyme digestion and all hits were sequenced for complete verification. The primers for DNA sequencing were universal primers or designed based on the sequences of P4-a/P5-a (see Table S6).

Computational section. In silico modeling and analysis. The 3D structures of designed proteins were predicted by using ColabFold based on protein sequences. Molecular docking of 2-

$HE(MHET)_3$ to PET hydrolases was performed using AutoDockTools 1.5.6. The energetically favorable orientations of ligands binding to the targeted sites of enzymes were extracted and analyzed, and the docking conformations were then visualized and analyzed by Pymol 2.5.4.

MD simulations. MD simulations and analysis were performed using the GROMACS 2018 simulation package[1] with the CHARMM36[2] force field. The designed proteins were solvated with 0.15 M NaCl solution in a cubic box with a minimal distance of 1.0 nm from the box edge to the protein. The water molecules are modeled with the TIP3P model. To avoid unfavorable interactions, energy minimization was done using the steepest descent method prior to MD simulations. After equilibration for 100 ps in NVT ensemble and 100 ps in NPT ensemble at 300 K, a production run of 20 and 100 ns was conducted at 1 bar and 300 K. All MD simulations were conducted with a time step of 2 fs. The coordinates, energy, and velocity were stored every 0.5 ns for trajectory analysis. RMSD (including the motif RMSD and time-dependent RMSD), SASA (solvent accessible surface area), and R_g (radius of gyration) were calculated with tools in Gromacs. Simulation trajectories were visualized and analyzed using Pymol 2.5.4 and VMD 1.9.3.

Inpainting and refining the new sequences to scaffold the functional motifs. After extracting the functional motifs from LCC, the missing sequences were completed by the RF_{joint}-guided inpainting approach. The retained amino acid sequences and structural information were used as input. During the inpainting, typically 10 repeated cycles were applied to generate high-quality inpainting sequences and structures. To further refine the inpainting results, we identified the problematic sequences (such as the SAAR and CHAA sequences) and large hydrophobic patches, and then deleted these regions. We utilized RF_{joint} to inpaint new sequences with the same length until there were no such flaws. Multiple rounds of inpainting were required to stepwise refine the sequences and structures.

Redesign of the protein sequence by ProteinMPNN. We used ProteinMPNN to redesign the entire sequences of the enzymes that exhibited PET hydrolytic activity. The open-source code of the ProteinMPNN algorithm is available on github. For each target backbone, we used the full protein backbone coordinates suggested by ColabFold as the input for ProteinMPNN to generate new sequences. We provided ColabFold-predicted protein structures along with all the corresponding amino acid sequences as input for global sequence optimization, without fixing the amino acids at any position. After 600 seconds of optimization, ProteinMPNN generated 10 distinct sequences for each target backbone (sampling temperature 0.1). The predicted structures of these sequences were

subsequently analyzed using ColabFold. After in silico screening via the computational approaches, the most promising sequences were selected for further evaluation.

The preparation of PET microparticles. The PET film was cut into 2 × 5 mm sized pieces and then crushed at room temperature using a high-speed rotary mill (8000 rpm). The microparticles obtained after the treatment was screened through a 50-mesh sieve plate to collect particles.

Differential Scanning Calorimetry (DSC). DSC profile measurements were done on a Perkin Elmer DSC2-00948 differential scanning calorimeter. The treated PET samples were dried in a vacuum desiccator at room temperature for 24 h. 5 mg of PET samples were taken and analysed by heating at a constant rate of 10 °C/min over a temperature range of 30-290 °C. PET crystallinity (Xc) was calculated according to Equation (1).

$$X_C = \frac{\Delta H_m - \Delta H_{cc}}{\Delta H_m^0} \times 100\% \qquad (1)$$

ΔH_{cc} is the enthalpy of cold crystallisation of the sample, ΔH_m is the enthalpy of melting of the sample, ΔH_m^0 is the enthalpy of melting of fully crystallised, with a value of ΔH_m^0 of 140.1 J/g.[3]

Enzymatic assays. The enzyme activity was assayed based on the concentrations of soluble hydrolysates released from hydrolytic reactions. When using BHET as the substrate, a certain amount of purified mixed proteins was added to 1 mL phosphate buffer (50 mM, pH 8.0) containing 1 mg/mL BHET to initiate the reaction. After reaction at 25 °C, 800 rpm for 24 h in an incubator, the hydrolysates were analyzed by HPLC (see below).

When using the PET microparticles as the substrate, enzymatic reactions were carried out with 6.0 μg/mL purified mixed proteins in the presence of 5 mg/ml of PET microparticles in phosphate buffer (50 mM, pH 8.0) at a given temperature under agitation at 800 rpm for 24 h. The reaction was quenched by heating to 100 ℃, followed by the addition of the same volume of 1‰ aqueous trifluoroacetic acid. The reaction mixture was then filtered with polyethersulfone syringe filters (0.2 μm in pore size) to remove the remained PET microparticles. The filtrate was analyzed using an HPLC system equipped with an Eclipse Plus C18 column. The mobile phase was composed of methanol and 1‰ aqueous trifluoroacetic acid, and the flow rate was 1.0 mL/min. The hydrolysates were monitored by a diode-array detection at a wavelength of 240 nm, and the temperature of the column oven was 40 °C.

The Michaelis-Menten kinetics of P4-a-2 and LCC was assayed with 0.03 μM enzyme and various PET loads at 50 ℃ in the shaking incubator at 800 rpm for 2 h. The concentrations of released hydrolysates were measured on a UV-Vis spectrophotometer at 240 nm, the products MHET, BHET,

and TPA were regarded as the TPA equivalents (TPA_{eq}), assuming having the same molar extinction coefficient of 17,000 $M^{-1}cm^{-1}$.[4] The K_m and k_{cat} values were determined by fitting the initial steady-state velocity to the Michaelis-Menten equation.

Size exclusion chromatography (SEC). AKTA pure M with UNICORN 6.3.2 Workstation control (GE Healthcare) coupled with a Superdex 75 Increase 10/300 GL column and buffer (10 mM Tris, 500 mM NaCl, 5 mM DTT) was used for size exclusion chromatography.

Differential scanning fluorescence (DSF). The protein sample solution was filled into 3 nanoDSF Grade Standard Capillaries (NanoTemper Technologies) and integrated in a Prometheus NT.48 device (Nanotemper Technologies) controlled by the PR.ThermControl software (version 2.1.2). Excitation power was pre-adjusted to get fluorescence readings above 2000 RFU for F330 and F350, and samples were heated from 40 °C to 80 °C with a slope of 0.5 °C/min. An XLSX file with "processed data" was exported from the PR.ThermControl software and used for further analysis.

Preparation of PET nanoparticles. 0.5 g of PET microparticles were dissolved in 25 mL of hexafluoro-2-propanol (HFIP). The solution was slowly poured into deionized water to form a stable PET suspension. The co-solvent HFIP in the suspension was removed by rotary evaporation at 47 °C, yielding PET nanoparticles dispersed in water. The size of the PET microparticles were measured by dynamic light scattering (DLS) on a Malvern Zetasizer Nano-ZS90 and transmission electron microscopy (TEM) on a JEM-1400Flash Electron Microscope. These characterizations showed that the PET particles in the suspension were 145 ± 50 nm.

Scanning electron microscopy (SEM). After cultivation, the films were washed with distilled water, and then ethanol. The morphology of PET films was examined by JSM7401 SEM (JEOL, Japan) at an accelerating voltage of 3 kV. PET films were sputter-coated with platinum in an ion sputter.

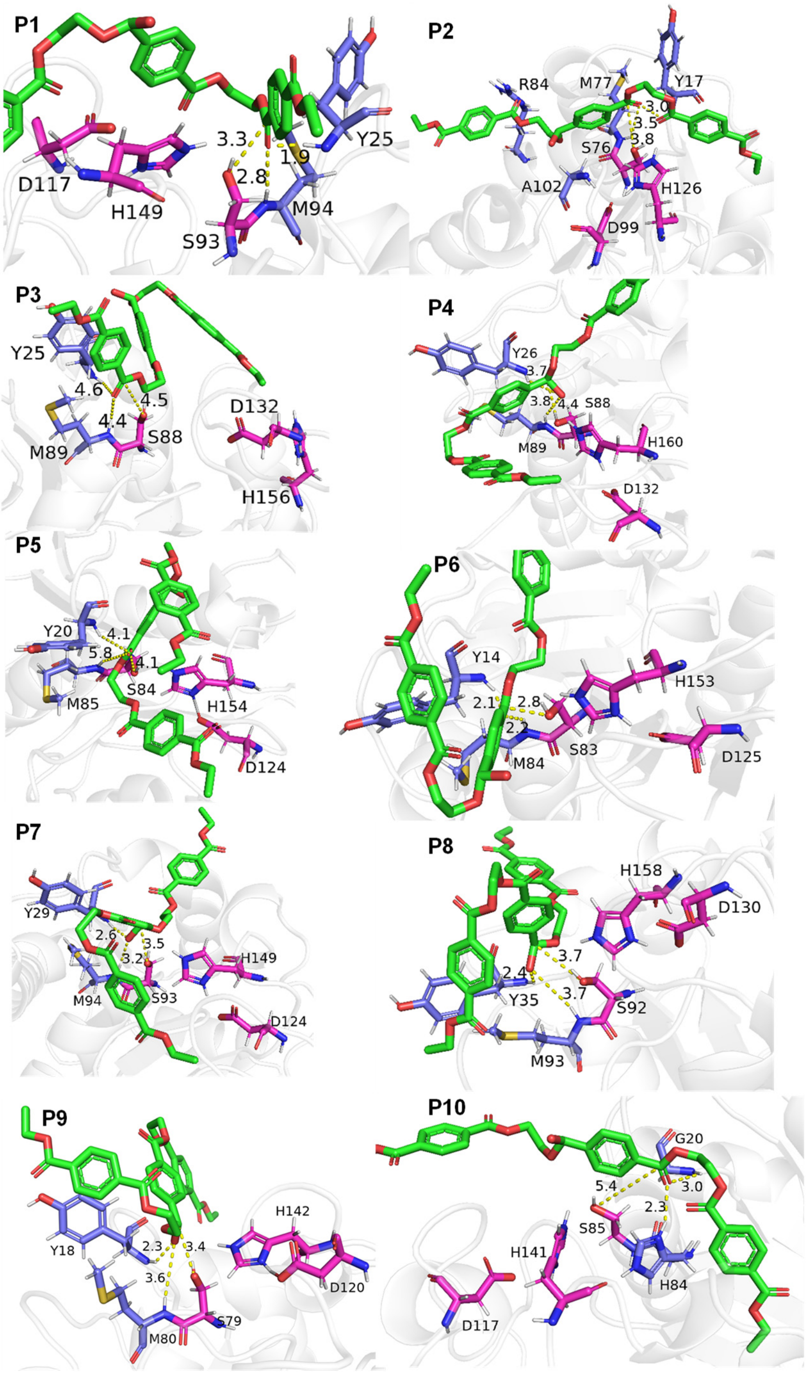

Fig. S1. Representative molecular docking of 2-HE(MHET)$_3$ (green stick model) with the virtual enzymes P1 to P10. The catalytic triad, Ser, His, and Asp are highlighted in magenta. The residues which can potentially form the oxyanion hole are colored in blue-grey. The distance between the oxygen atom from the side chain of the Ser residue and the carbonyl carbon of the ester bond is indicated in each docking with dashed lines. Oxygen atoms are colored in red, hydrogen atoms in white, nitrogen atom in blue, and sulphur atoms in yellow.

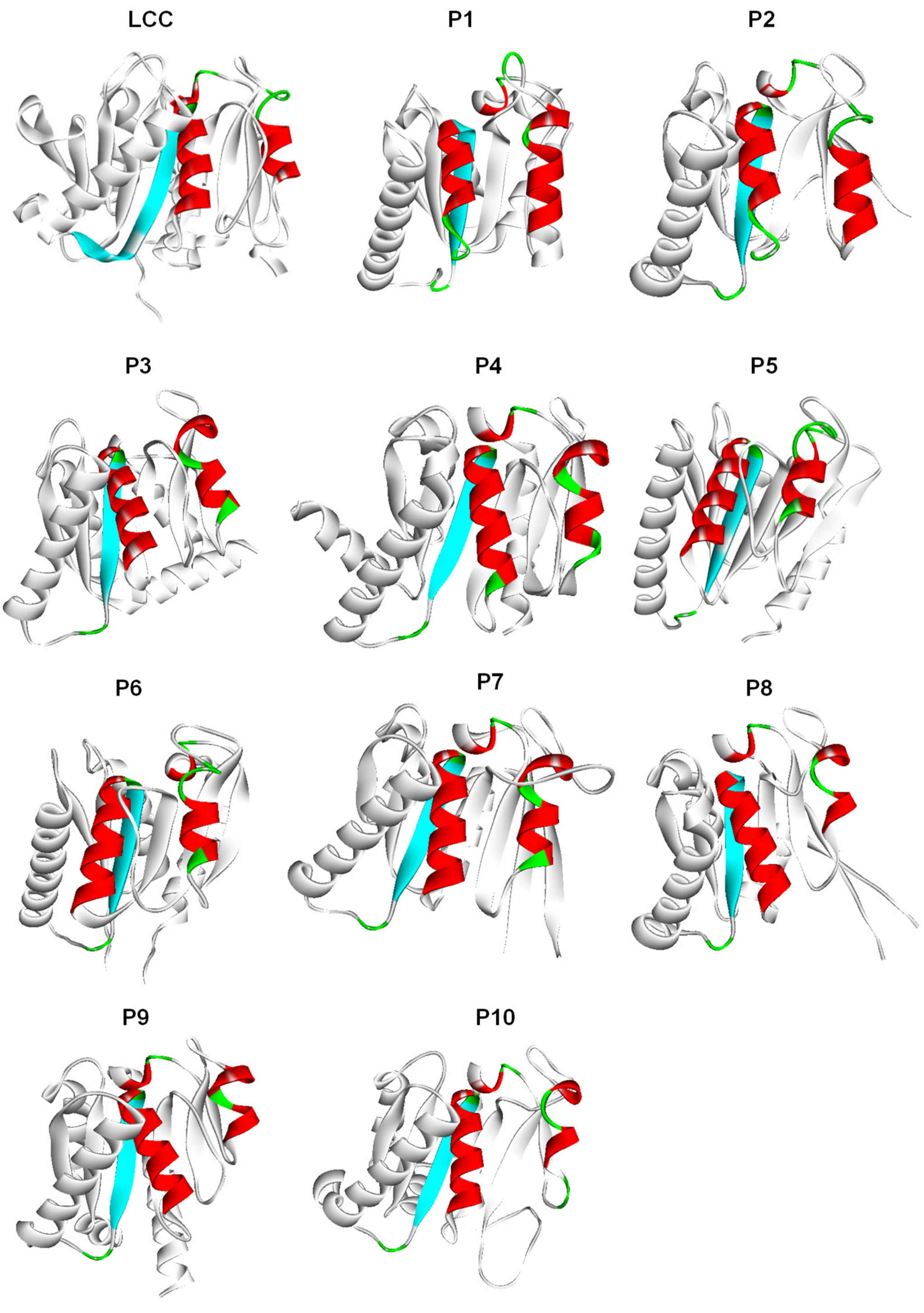

Fig. S2. The predicted 3D structures of LCC and the 10 virtual enzymes generated in the first round of design. The newly generated protein scaffolds are colored in gray, while the retained regions from the template enzyme LCC are colored in red, green and blue.

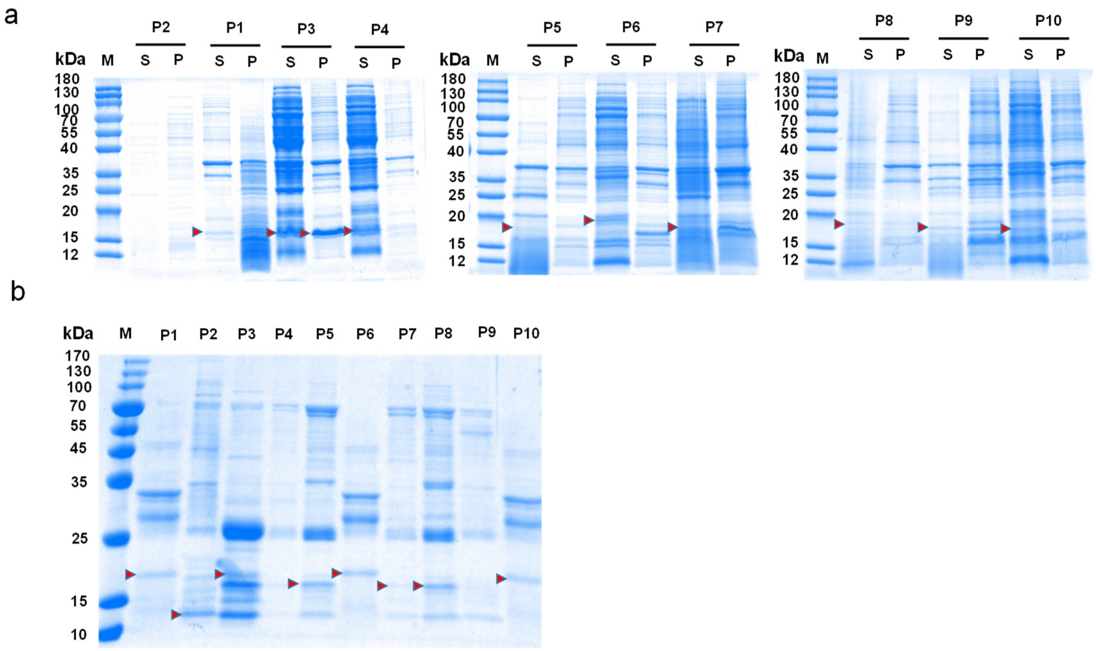

Fig. S3. a, SDS-PAGE analysis of the expression of P1–P10 by *E. Coli* BL21 (DE3) cells, S represents the soluble fraction of the cell lysate, P represents the precipitates of the cell lysate. Only P3 formed inclusion bodies. **b,** SDS-PAGE analysis of P1–P10 after purification by Ni-NTA chromatography. The expected bands of target proteins are indicated with the red arrows. Although the bands of P1, P2, P3, P5, P6, P7, P8, P10 are shown in the gel, their expression levels are too low to be further purified.

a

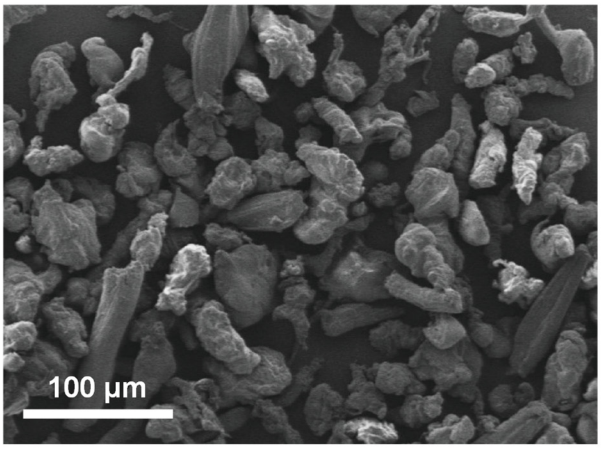

b

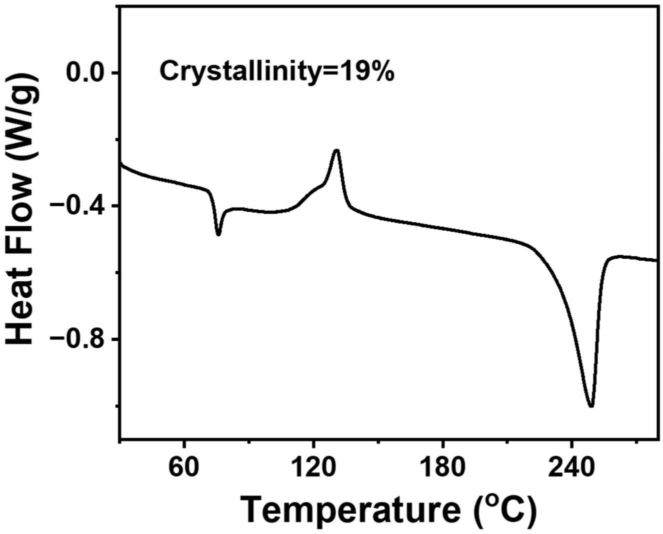

Fig. S4. a, SEM images and **b,** DSC analysis of PET microparticles.

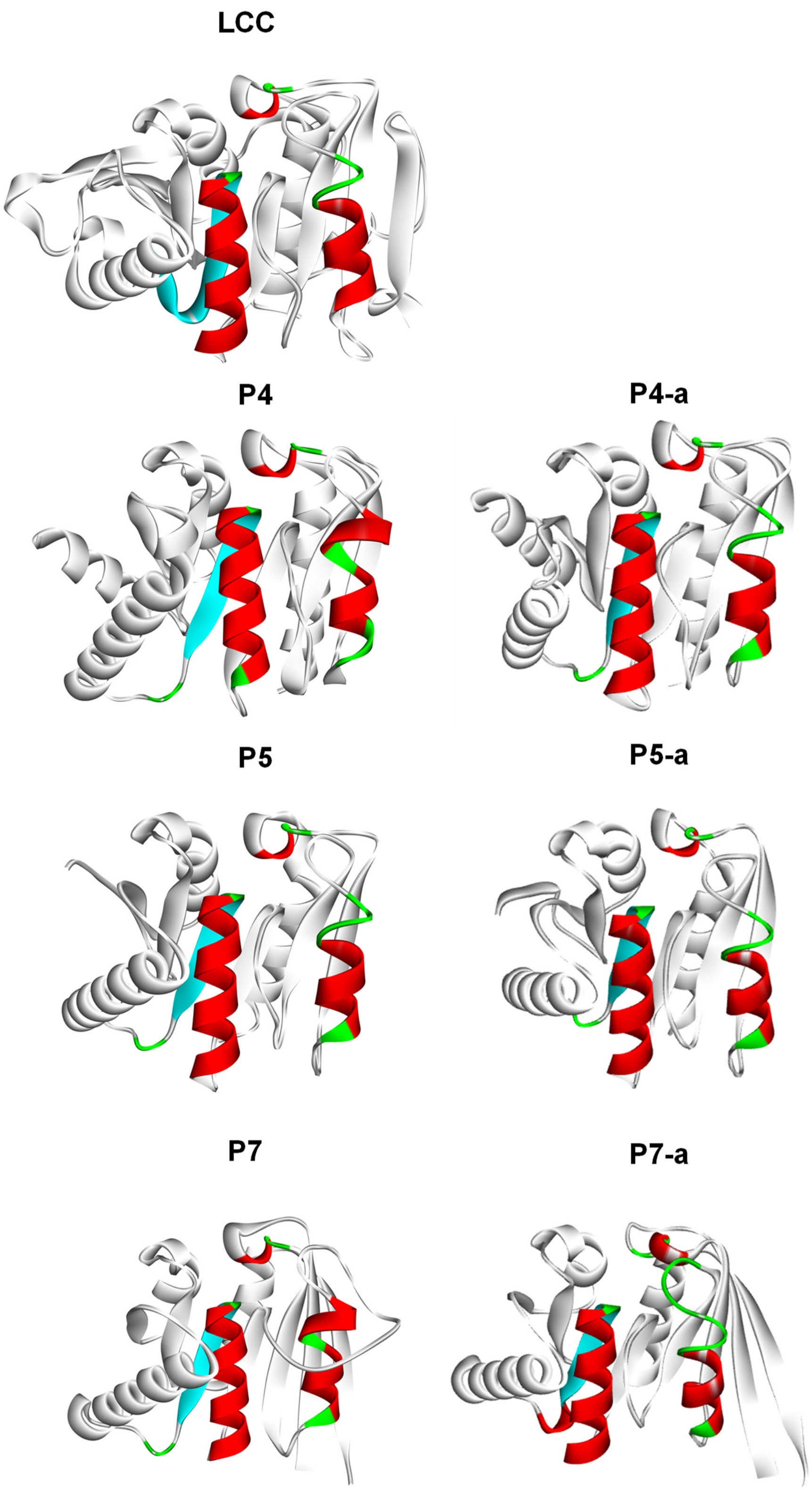

Fig. S5. The 3D structures of LCC and the redesigned enzymes using iterative RF_{joint}. The regenerated protein scaffolds are colored in grey while the retained regions from the template enzyme LCC are colored in red, green and blue.

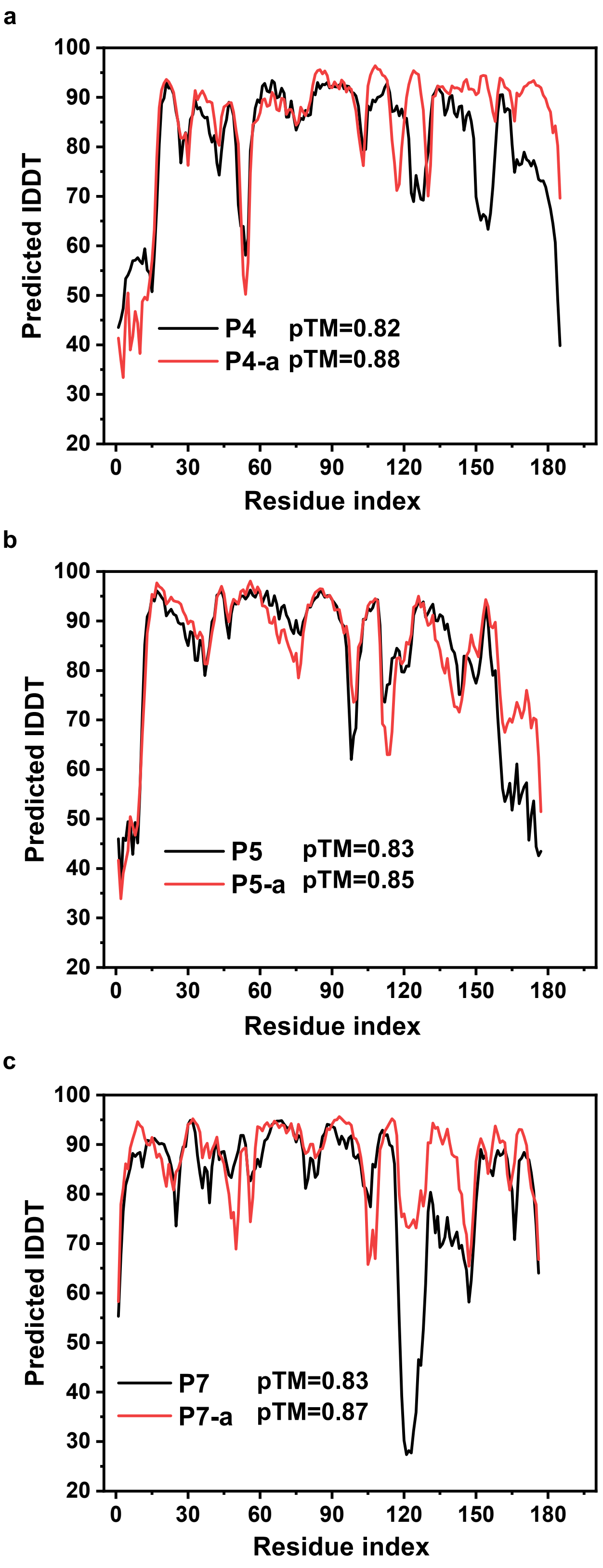

Fig. S6. Comparisons of the pLDDT values and pTM-scores before and after sequence refinement by RF_{joint} inpainting. **a,** P4 (black curve) and P4-a (red curve); **b,** P5 (black curve) and P5-a (red curve); (c) P7 (black curve) and P7-a (red curve).

a

```
LCC               1 SNPYQRGPNPTRSALTADGPFSVATYTVSRLSVSGFGGGVIYYPTGTSLT    50
                    |       |..|:...|:              |...|.|.:
P4-a              1 S-------NAERARALAE--------------VRARGRGAL---------    20

LCC              51 FGGIAMSPGYTADASSLAWLGRRLASHGFVVLVINTNSRFDYPDSRASQL   100
                        .:..||||||||||||||||||. |.||::|..    |||||||||
P4-a             21 ----VLDGGYTADASSLAWLGRRLASL-FGVLLVNAR----YPDSRASQL    61

LCC             101 SAALNYLRTSSPSAVRARLDANRLAVAGHSMGGGGTLRIAEQNPSLKAAV   150
                    |||||||||...|   ..|||||||||||||||||||||||||......:
P4-a             62 SAALNYLRTQLAS---LGLDANRLAVAGHSMGGGGTLRIAEQNRGRPRVL   108

LCC             151 PLTPWHTDKTFNTSVPVLIVGAEADTVAPVSQHAIPFYQNLPSTTPKVYV   200
                    ..|||.:.........|| .|..:|||||||||||||||||.....:|..
P4-a            109 AFTPWESGPARGGRADVL-AGRGSDTVAPVSQHAIPFYQNLRGGRLRVLP   157

LCC             201 ELDNASHFAPNSNNAAISVYTISWMKLWVDNDTRYRQFLCNVNDPALSDF   250
                        |.|||||..:.|:.....:.::.|...
P4-a            158 ----APHFAPNRGSPAVVAAARAALEAWEAQ-------------------   184

LCC             251 RTNNRHCQ    258
                           |
P4-a            185 -------Q    185
```

b

```
LCC               1 SNPYQRGPNPTRSALTADGPFSVATYTVSRLSVSGFGGGVIYYPTGTSLT    50
                    ||.         ..|.|....::..|.
P5-a              1 SNA---------QLLRAGAQLALVLYA-----------------------    18

LCC              51 FGGIAMSPGYTADASSLAWLGRRLASHGFVVLVINTNSRFDYPDSRASQL   100
                            ||||||||||||||||||.|..||:::...   |||||||||
P5-a             19 --------GYTADASSLAWLGRRLASLGLAVLLLSGGG---YPDSRASQL    57

LCC             101 SAALNYLRTSSPSAVRARLDANRLAVAGHSMGGGGTLRIAEQNPSLKAAV   150
                    |||||||||...|   ..|||||||||||||||||||||||||.....|:
P5-a             58 SAALNYLRTQLAS---LGLDANRLAVAGHSMGGGGTLRIAEQNRGGLLAL   104

LCC             151 PLTPWHTDKTFNTSVPVLIVGAEADTVAPVSQHAIPFYQNLPSTTPKVYV   200
                    ..|||...:....   ||::|  :|||||||||||||||||.....::..
P5-a            105 AFTPWEGGRAGRA---VLVLG--SDTVAPVSQHAIPFYQNARPGGFRLLR   149

LCC             201 ELDNASHFAPNSNNAAISVYTISWMKLWVDNDTRYRQFLCNVNDPALSDF   250
                    ...|  |||||:..|.                            .||.:.
P5-a            150 VPGN--HFAPNNGRAL----------------------------DALEEL   169

LCC             251 RTNNRHCQ    258
                    ....:..|
P5-a            170 LQLLQQQQ    177
```

c

```
LCC               1 SNPYQRGPNPTRSALTADGPFSVATYTVSRLSVSGFGGGVIYYPTGTSLT    50
                    |       |.:......|....:|.:....|.:..
P7-a              1 S-------NCSVEVRRLDSLEELADFLEEELGLDD---------------    28

LCC              51 FGGIAMSPGYTADASSLAWLGRRLASHGFVVLVINTNSRFDYPDSRASQL   100
                       :|::.||||||||||||||||||     |::|.|.   |||||||||
P7-a             29 ---VALASGYTADASSLAWLGRRLAS-----LLVNGNG---YPDSRASQL    67

LCC             101 SAALNYLRTSSPSAVRARLDANRLAVAGHSMGGGGTLRIAEQNPSLKAAV   150
                    |||||||||:.|.     ||||||||||||||||||||||||| .|..|:
P7-a             68 SAALNYLRTNRPG-----LDANRLAVAGHSMGGGGTLRIAEQN-GLPGAL   111

LCC             151 PLTPWHTDKTFNTSVPVLIVGAEADTVAPVSQHAIPFYQNLPSTTPKVYV   200
                    ..|||..    .....:|::|  |||||||||||||||||||...    :
P7-a            112 VFTPWGP----GGGGRLLLLG--ADTVAPVSQHAIPFYQNLPGGR----L   151

LCC             201 ELDNASHFAPNSNNAAISVYTISWMKLWVDNDTRYRQFLCNVNDPALSDF   250
                    .|....|||||:....:.:..::
P7-a            152 ALSGDGHFAPNNGRGRVLLLEVT---------------------------   174

LCC             251 RTNNRHCQ    258
                          ||
P7-a            175 ------CQ    176
```

Fig. S7. Pairwise sequence alignment of the newly designed enzymes with respect to the template enzyme LCC using the online tool provided by EMBL-EBI.[5] The sequence identity of P4-a, P5-a and P7-a with LCC is 41%, 40% and 40%, respectively. The sequence similarity of P4-a, P5-a and P7-a with LCC is 47%, 46% and 47%, respectively.

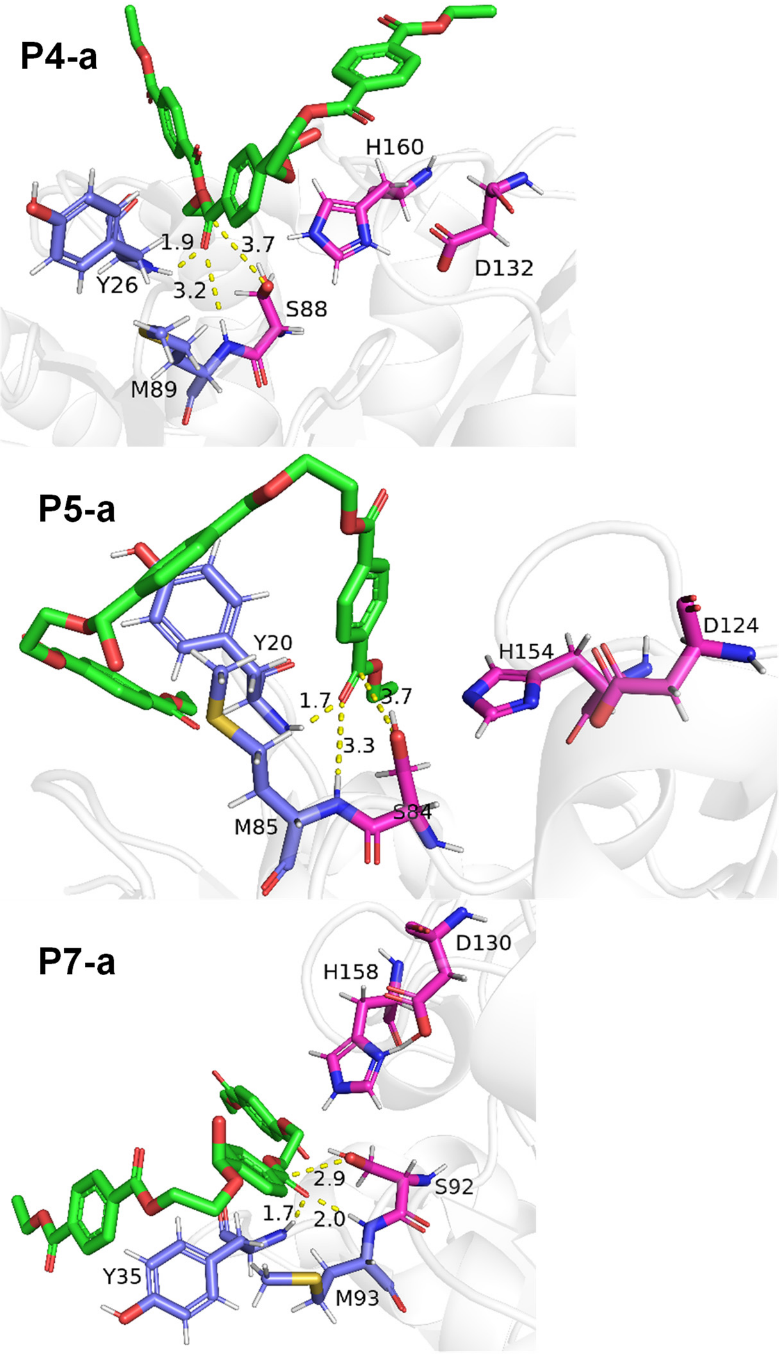

Fig. S8. Representative molecular docking of 2-HE(MHET)$_3$ (green colored stick model) with the virtual enzymes P4-a, P5-a and P7-a. The catalytic residues (Ser-His-Asp) are highlighted in magenta. The residues which can potentially form the oxyanion hole are colored in blue-grey. The distance between the oxygen atom from the side chain of the Ser residue and the carbonyl carbon of the ester bond is indicated in each docking with dashed lines. Oxygen atoms are colored in red, hydrogen atoms in white, nitrogen atom in blue, and sulfur atoms in yellow.

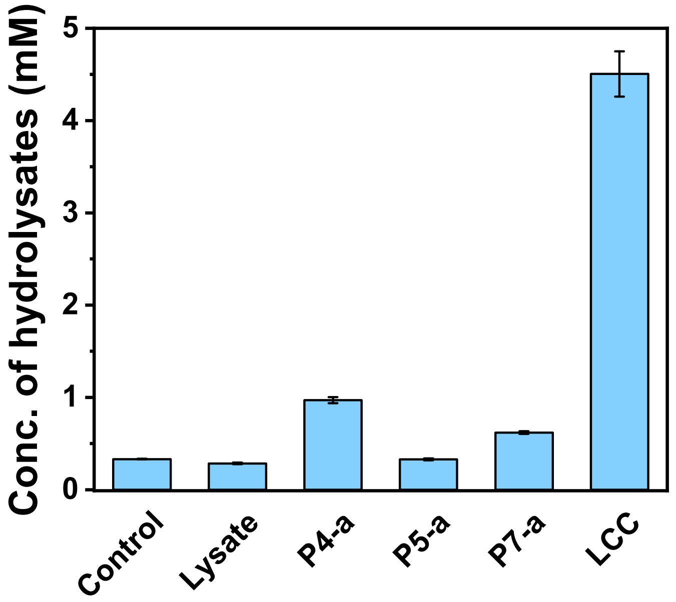

Fig. S9. The hydrolysis of BHET by the eluted protein fractions from a Ni-NTA affinity column. The reaction system contained 6.0 μg/mL proteins and 1.0 mg/mL BHET and was incubated at 25 °C for 24 h. "Control" represents the self-hydrolysis of BHET at the same conditions, and "Lysate" represents the BHET hydrolysis by the cell lysate of *E. coli* carrying empty plasmids. The concentrations of hydrolysates were determined by HPLC. Error bars represent the standard deviations of three measurements.

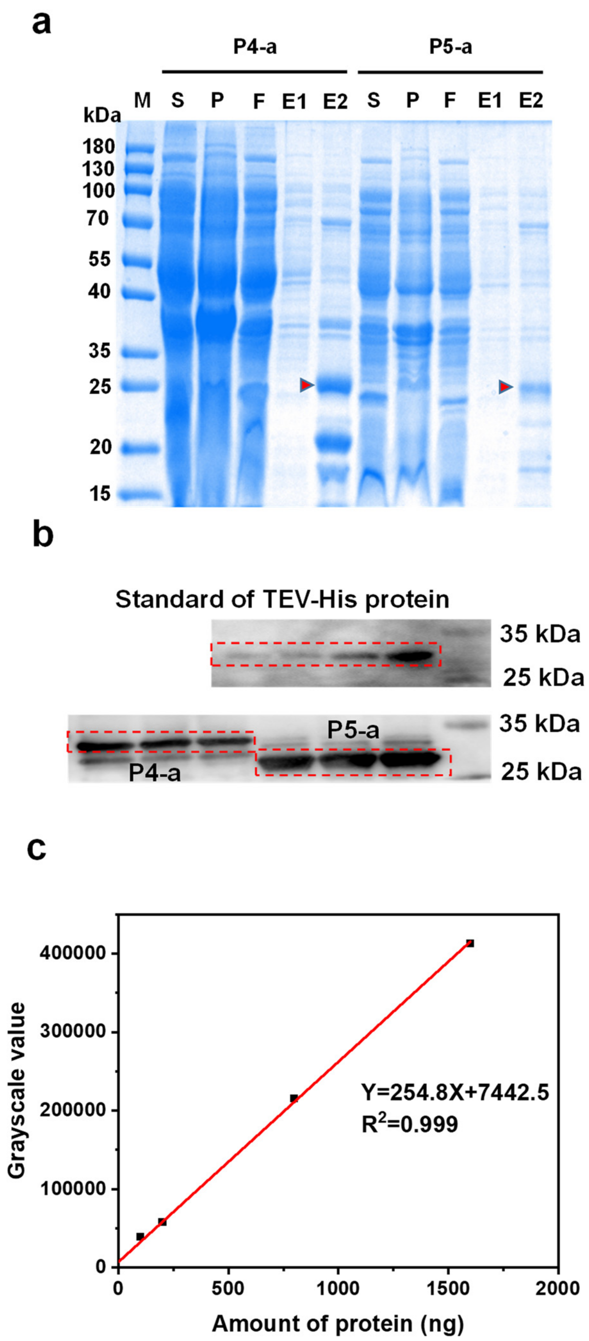

Fig. S10. a, SDS-PAGE analysis of the expression of His-tagged P4-a and P5-a in *E. Coli BL21 (DE3) PLysS* cells. S represents the soluble fraction of the cell lysate, P represents the precipitates of the cell lysate, F represents the flow-through fraction of the cell lysate, E1 represents the eluted by 50 mM imidazole and E2 represents the eluted by 300 mM imidazole from Ni-NTA affinity chromatography. **b,** Western blotting analysis of the His-tagged P4-a, P5-a. TEV-His protein was used as the protein standard for concentration assay. The proteins were visualized by anti-His6 western blotting. **c,** Standard curve (grey value as a function of protein amount) calibrated with TEV protein.

a

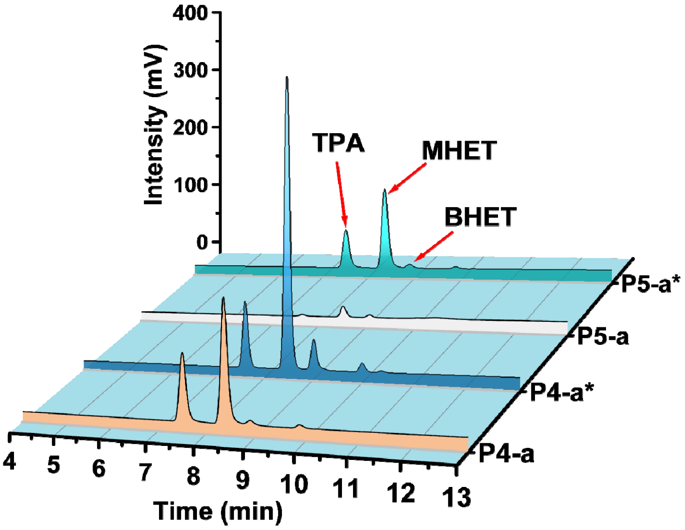

b

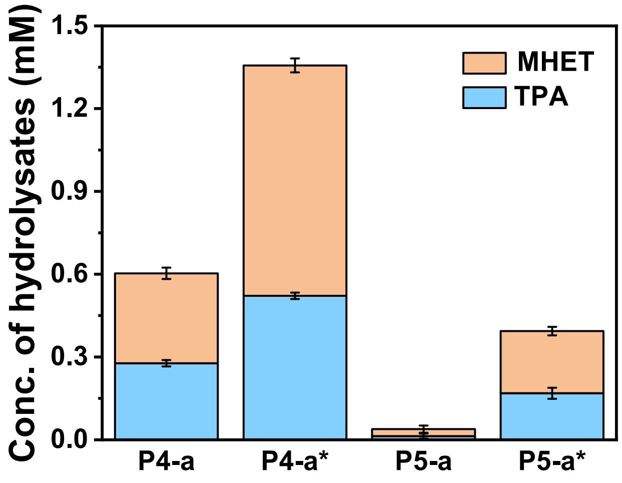

Fig. S11. Hydrolysis of PET microparticles by P4-a and P5-a expressed with or without SUMO fusion. **a,** the HPLC chromatograms and **b,** the concentrations of hydrolysates released by P4-a and P5-a expressed in different ways. P4-a and P5-a were expressed directly. P4-a* and P5-a* were expressed with SUMO tags, which were removed by adding ULP1 protease. As the expression levels were still too low to obtain pure proteins. All reactions were carried out by the eluted protein fractions after Ni-NTA chromatography. The reaction system contained 6.0 μg/mL proteins and 5 mg/ml of PET microparticles and was incubated at 60 °C for 24 h. The concentrations of hydrolysates BHET, MHET and TPA were determined by HPLC.

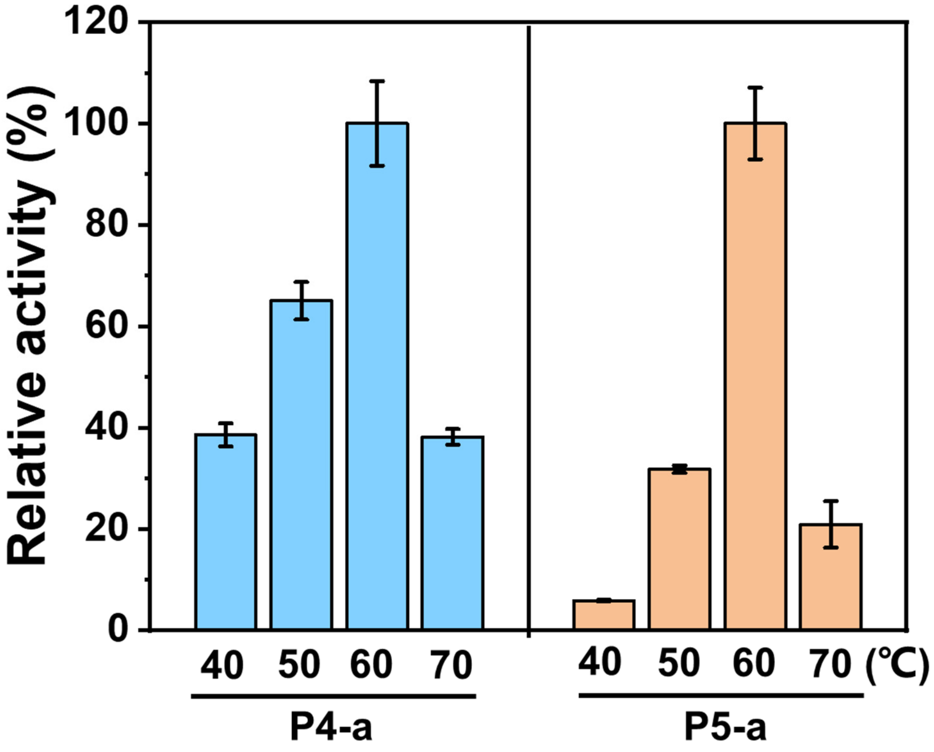

Fig. S12. The activity-temperature dependence of P4-a and P5-a. All assays were carried out with 5 mg/ml of PET microparticles in phosphate buffer (50 mM, pH 8.0). The concentrations of hydrolysates BHET, MHET and TPA were determined by HPLC. Error bars represent the standard deviations of three measurements.

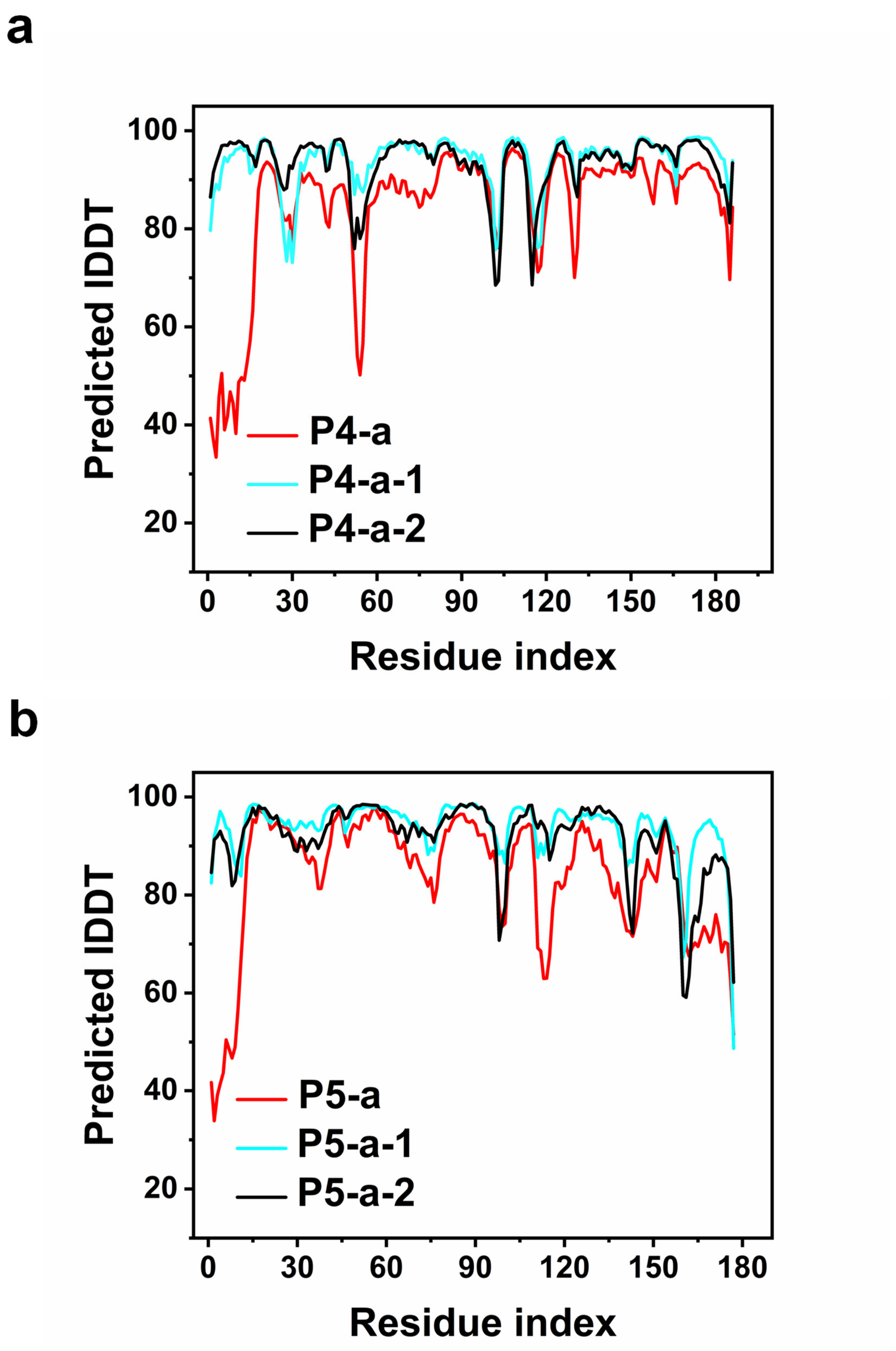

Fig. S13. Comparisons of the pLDDT values before and after ProteinMPNN rescue. **a,** P4-a (red curve), P4-a-1 (blue curve) and P4-a-2 (black curve); **b,** P5-a (red curve), P5-a-1 (blue curve) and P5-a-2 (black curve).

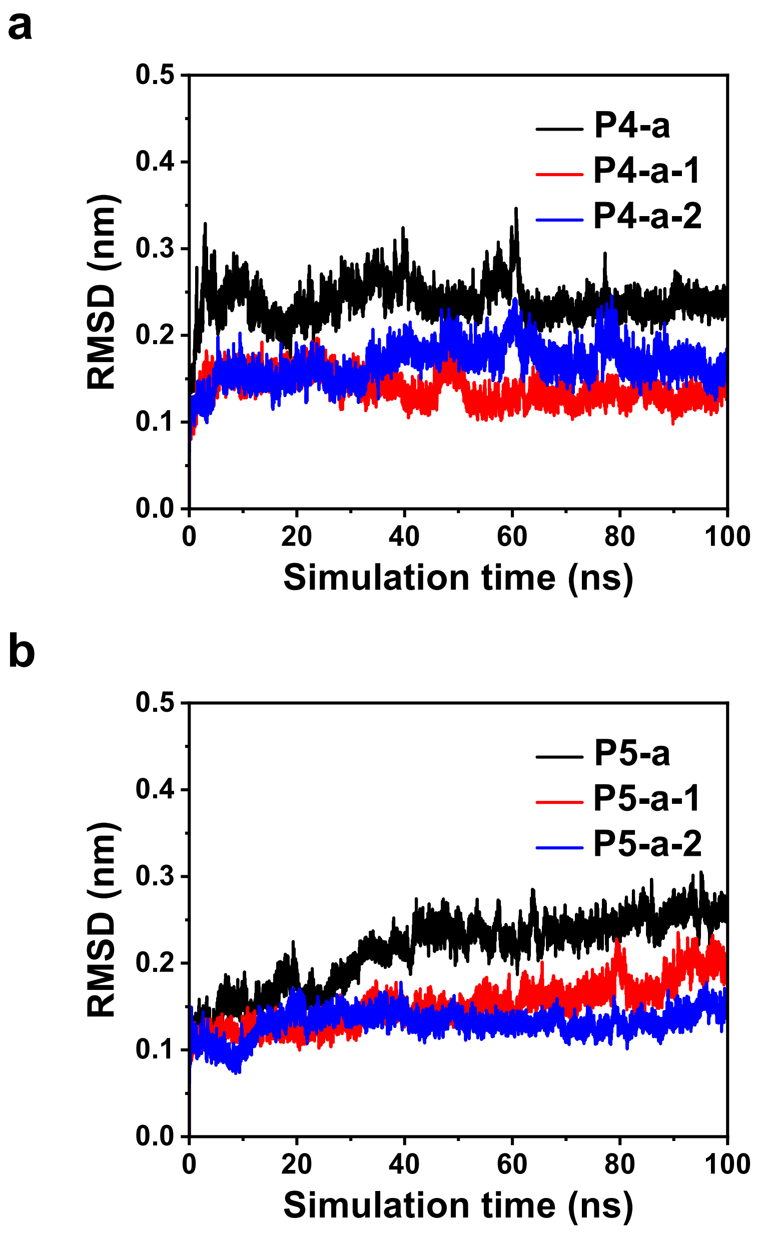

Fig. S14. Time-course RMSD fluctuations of **a,** P4-a, P4-a-1 and P4-a-2; and **b,** P5-a, P5-a-1 and P5-a-2.

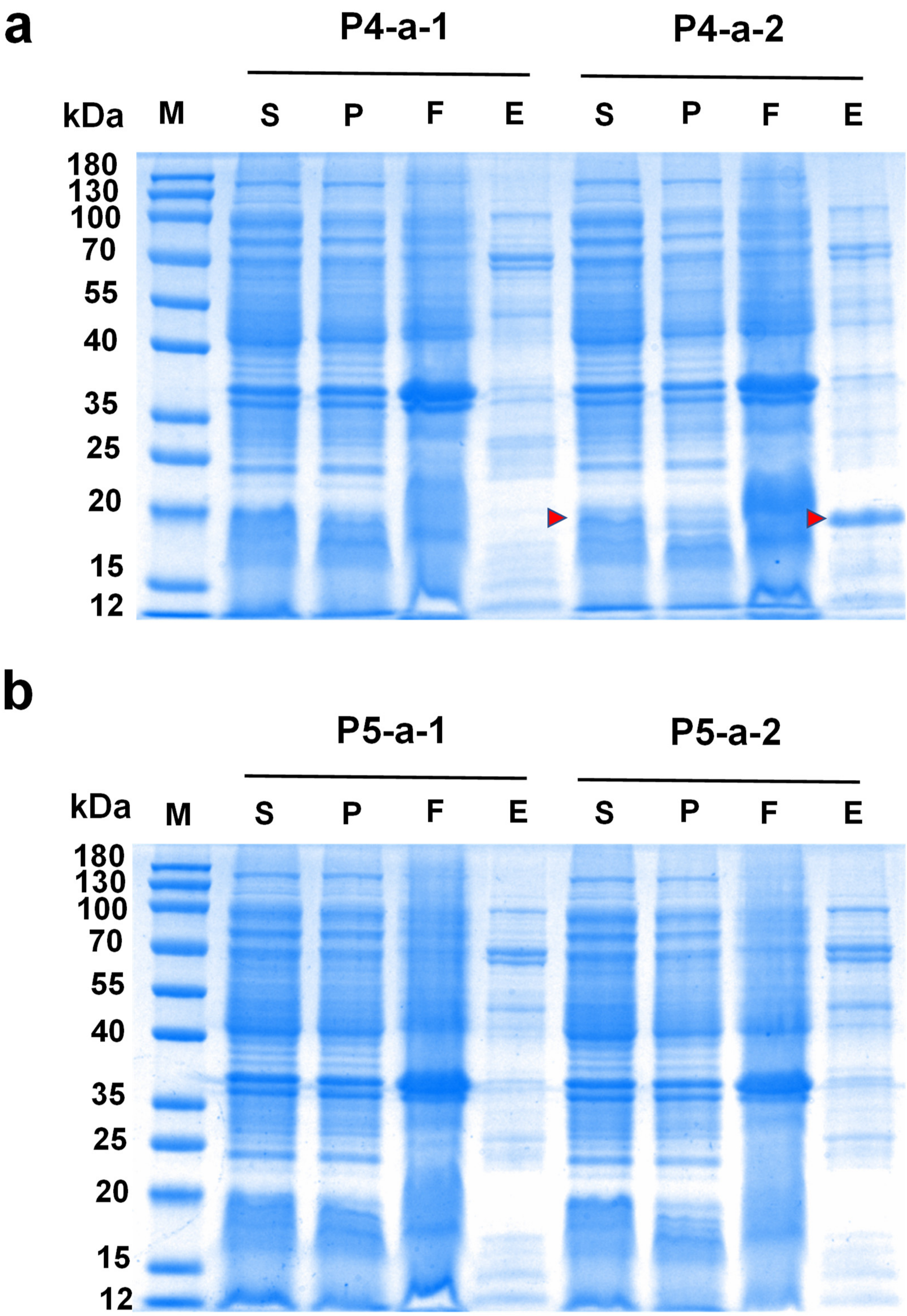

Fig. S15. SDS-PAGE plot of Coomassie brilliant blue stained numbered P4-a-1, P4-a-2, P5-a-1 and P5-a-2 proteins. S represents the soluble fraction of the cell lysate, P represents the precipitates of the cell lysate, F represents the flow-through fraction of the cell lysate, E represents the eluted proteins from the Ni-NTA affinity chromatography.

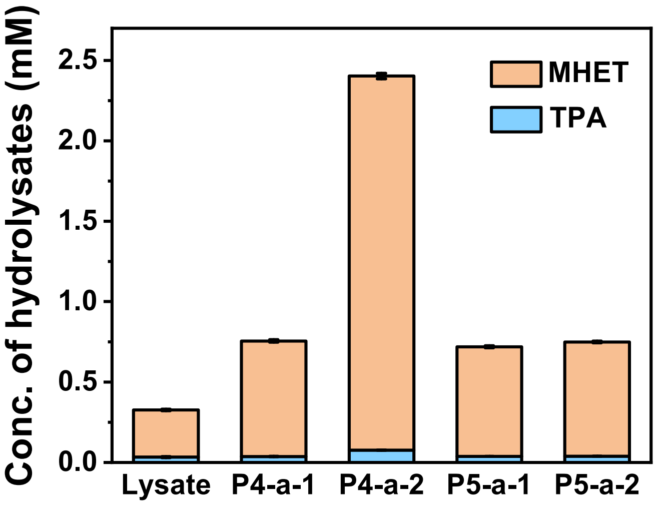

Fig. S16. The hydrolysis of BHET by the ProteinMPNN rescued enzymes (assayed with the eluted fractions from the Ni-NTA affinity column). Conditions: 6.0 μg/mL eluted proteins, 1.0 mg/mL BHET, 25 ^{o}C for 24 h. "Lysate" represents the BHET hydrolysis by the cell lysate of *E. coli* carrying empty plasmids. The hydrolytic products TPA and MHET were quantified by HPLC. Error bars represent the standard deviations of three measurements.

a

```
LCC            1 SNPYQRGPNPTRSALTADGPFSVATYTVSRLSVSGFGGGVIYYPTGTSLT     50
                 :.                     |.:..:...:...|.|.:
P4-a-2         1 TE---------------------AEWARALAQLRALGRGAL---------     20

LCC           51 FGGIAMSPGYTADASSLAWLGRRLASHGFVVLVINTNSRFDYPDSRASQL    100
                     .::.||.....:||.||..|:....|:|:...:     |:..|..:
P4-a-2        21 ----VLNSGYGESPEALAGLGEALSELLGVLLIAAPD-----PEKAADDI     61

LCC          101 SAALNYLRTSSPSAVRARLDANRLAVAGHSMGGGGTLRIAEQNPSLKAAV    150
                 .|||..|   :.:.....||...|||.|||:|||.||.:||::|.....:
P4-a-2        62 EAALARL---AAALAALGLDPRLLAVLGHSLGGGATLEVAERHPGWPLVI    108

LCC          151 PLTPWHTDKTFNTSVPVLIVGAEADTVAPVSQHAIPFYQNLPSTTPKVYV    200
                 .|.|. ..:........:|.|..|||:||..::|:|.|    :......:
P4-a-2       109 ALAPL-APRVARGGTADIIGGVGADTIAPYEEYALPHY----AAADGGRL    153

LCC          201 ELDNASHFAPNSNNAAISVYTISWMKLWVDNDTRYRQFLCNVNDPALSDF    250
                 .|....|||....:.|:.....:.::.|:..
P4-a-2       154 TLLPLPHFAATQGSPAVIAAVKAAVEAWLAT-------------------    184

LCC          251 RTNNRHCQ    258
                        |
P4-a-2       185 -------Q    185
```

b

```
ISPETase       1 MNFPRASRLMQAAVLGGLMAVSAAATAQTNPYARGPNPTAASLEASAGPF     50
                 .....|..|.|...||                 ||    |..|.
P4-a-2         1 TEAEWARALAQLRALG-----------------RG----ALVLN------     23

ISPETase      51 TVRSFTVSRPSGYGAGTVYYPTNAGGTVGAIAIVPGYTARQSSIKWWGPR    100
                           ||||..    |....|...|::.:.|.
P4-a-2        24 ----------SGYGES----PEALAGLGEALSELLGV-------------     46

ISPETase     101 LASHGFVVITIDTNSTLDQPSSRSSQQMAALRQVASLNGTSSSPIYGKVD    150
                      .::...|.....|...:..::..|||..:.             :|
P4-a-2        47 -----LLIAAPDPEKAADDIEAALARLAAALAALG-------------LD     78

ISPETase     151 TARMGVMGWSMGGGGSLISAANNPSLKAAAPQAPWDSSTNFSSVTVPTLI    200
                 ...:.|:|.|:|||.:|..|..:|........||  .:...:......:|
P4-a-2        79 PRLLAVLGHSLGGGATLEVAERHPGWPLVIALAP--LAPRVARGGTADII    126

ISPETase     201 FACENDSIAPVNSSALPIYDSMSRNAKQFLEINGGSHSCANSGNSNQALI    250
                 .....|:|||....|||.|.:........|.:   .|..|..|:......
P4-a-2       127 GGVGADTIAPYEEYALPHYAAADGGRLTLLPL---PHFAATQGSPAVIAA    173

ISPETase     251 GKKGV-AWMKRFMDNDTRYSTFACENPNSTRVSDFRTANCS    290
                 .|..| ||:..                            .
P4-a-2       174 VKAAVEAWLAT----------------------------Q    185
```

Fig. S17. Pairwise sequence alignment of the P4-a-2 enzyme with respect to **a,** LCC and **b,** *Is*PETase. The sequence identity and similarity of P4-a-2 compared to LCC is 21% and 34%, respectively. The sequence identity and similarity of P4-a-2 compared to *Is*PETase is 18% and 25%, respectively.

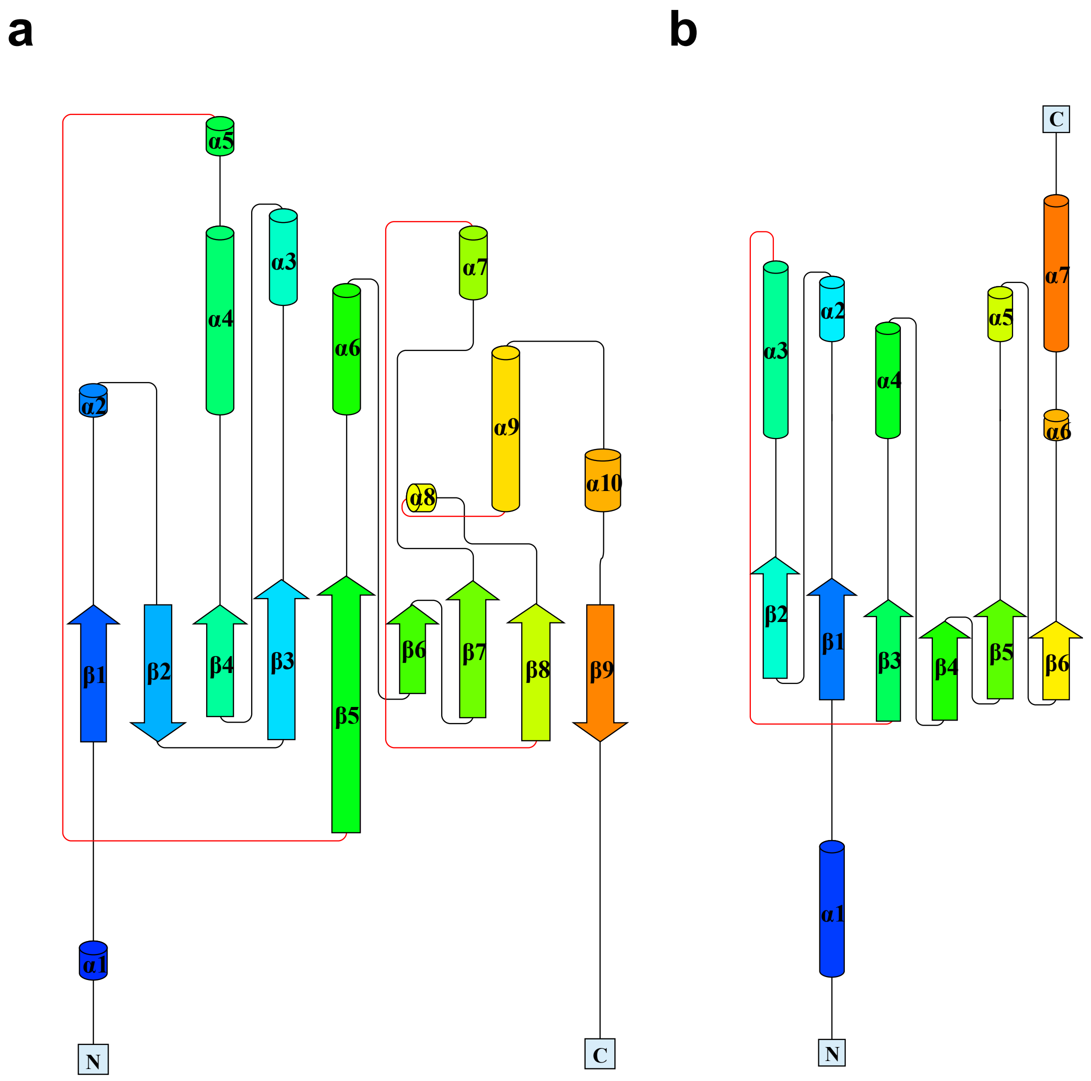

Fig. S18. The topology and ribbon diagrams of **a,** LCC. **b,** *RS*PETase 1. The coloring in the topology diagram from blue to red indicates the N- to C-terminal position.[6]

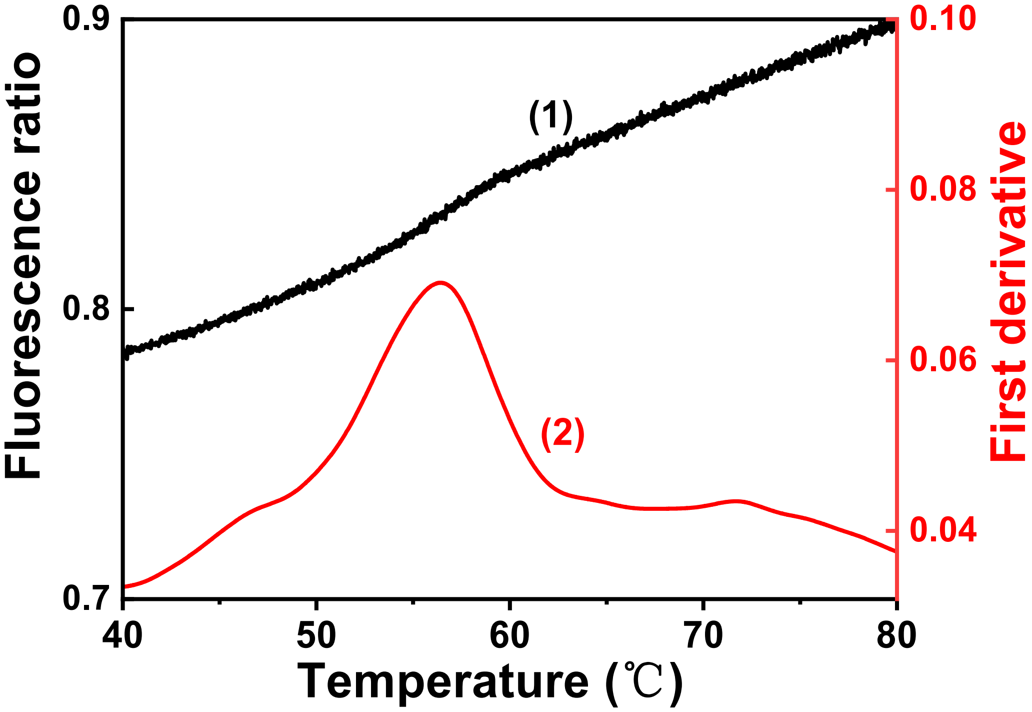

Fig. S19. DSF scanning of *Rs*PETase 1. The black curve represents the ratio of intrinsic fluorescence (350 nm: 330 nm) in DSF, and the red curve shows the first derivative.

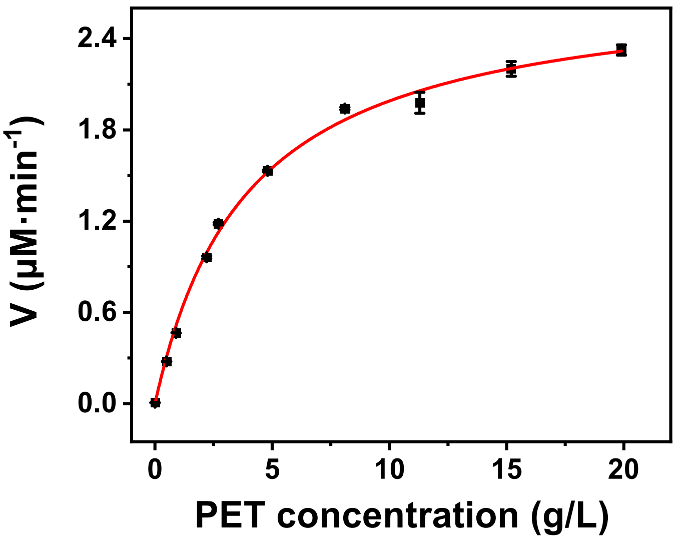

Fig. S20. Michaelis-Menten plot of LCC assayed at 50 °C.

Table S1: Detailed information of the designer enzymes P1-P10, P4-a, P5-a and P7-a.

The molecular weight, amino acid length, motif RMSD compared to the retained template structures by VMD 1.9.3, pLDTT and TM-score given by AlphaFold 2, the size and depth of the active pocket, and calculated binding energy with 2-HE(MHET)$_3$ are summarized below.

Protein	Molecular Weight (kDa)	Length (aa)	Binding energy (kcal/mol)	Motif RMSD (Å)	pLDDT	Volume (Å^3) *	Depth (Å) *
LCC	27.78	258	-2.68	-	-	245	13.8
P1	16.26	160	-3.57	1.81	71.32	191	9.2
P2	14.28	140	-2.67	2.13	70.62	534	14.9
P3	18.06	176	-0.82	2.00	79.72	80	6.7
P4	19.24	185	-2.3	1.74	80.50	213	11.7
P5	18.17	177	-1.49	1.05	82.34	222	13.8
P6	19.31	180	-1.84	1.29	89.79	221	13.1
P7	17.93	176	-1.74	1.32	81.89	201	10.6
P8	17.22	169	-2.31	2.06	74.76	222	13.3
P9	17.82	173	-1.67	2.22	83.14	249	15.3
P10	18.68	186	-3.66	1.92	61.64	103	6.8
P4-a	19.43	185	-2.74	1.22	84.39	215	11.8
P5-a	18.47	177	-1.18	1.05	83.72	252	13.1
P7-a	18.34	176	-2.57	1.11	86.92	221	12.6

* The size of active pocket and substrate binding energy were calculated by Protein plus.[7]

Table S2. Amino acid sequences of the wild-type LCC and designer enzymes.

The underlined sequences are retained for computational rescaffolding, and the catalytic triad is highlighted in red.

Wild-type LCC

SNPYQRGPNPTRSALTADGPFSVATYTVSRLSVSGFGGGVIYYPTGTSLTFGGIAMSPGYTA

DASSLAWLGRRLASHGFVVLVINTNSRFDYPDSRASQLSAALNYLRTSSPSAVRARLDAN

RLAVAGHSMGGGGTLRIAEQNPSLKAAVPLTPWHTDKTFNTSVPVLIVGAEADTVAPVSQ

HAIPFYQNLPSTTPKVYVELDNASHFAPNSNNAAISVYTISWMKLWVDNDTRYRQFLCNV

NDPALSDFRTNNRHCQ

Amino acid sequences of designer enzymes

> P1

SNTLGLGLELALLRGAGALALAGGYTADASSLAWLGRRLASELGLAVALVPAGGYPDSR

ASQLSAALNYLRTRLAARGLSRLDANRLAVAGHSMGGGGTLRIAEQGLLLALTPWGGDT

VAPVSQHAIPFYQNLLRLGLRGVVLVVGGNHFAPNNGLGLCQ

> P2

SNSLDGLGDAFALAAGYTADASSLAWLGRRLASLLVPAGYPDSRASQLSAALNYLRTQLA

SELGLDANRLAVAGHSMGGGGTLRIAEQGLLLVLTPWGDTVAPVSQHAIPFYQNCGLPLL

LAGPGHFAPNNGLGLGLNCQ

> P3

SNAALLLALLAGLRQARALALASGYTADASSLAWLGRRLASELGLGVLLVNGNGYPDSR

ASQLSAALNYLRTNRPGLDANRLAVAGHSMGGGGTLRIAEQNGLPVALAFTPWEPGPPRG

GLLLLLLGLGGGDTVAPVSQHAIPFYQNSGLGLALPHFAPNNGRAFEALEELLAQCQ

> P4

SNALLLLLLLLLLLGGGLLLALAGGYTADASSLAWLGRRLASLFGVLLLSGGYPDSRASQ

LSAALNYLRTQLASLGLDANRLAVAGHSMGGGGTLRIAEQNRGGLLVLVFTPWESGPARG

GRLVVLVVGASDTVAPVSQHAIPFYQNLRGGLVVLVGLPHFAPNRGSPELLEALLRLLRLL

LQCQ

> P5

SNLLLLLLLGAGALALYAGYTADASSLAWLGRRLASLGLAVLLLSGGGYPDSRASQLSAA

LNYLRTQLASLGLDANRLAVAGH**S**MGGGGTLRIAEQNRGGLLALAFTPWEGGRAGRAVL

VLGS**D**TVAPVSQHAIPFYQNARPGGGLLLLLGGN**H**FAPNNGRALDALEELLQLLQQCQ

> P6

SNPRPRLLALASGYTADASSLAWLGRRLASLFPNLGVALVDGRNEYPDSRASQLSAALNY

LRTLAEAQGLGLDANRLAVAGH**S**MGGGGTLRIAEQNGVPFVVFTPWDAEPPRGGRLLLV

VGGRN**D**TVAPVSQHAIPFYQNNPNLRLVLLPGN**H**FAPNDPELLEELLELLLELLLLLPQCQ

> P7

SNCSLLLLLLSDLEELLELLEALLGGLAFALASGYTADASSLAWLGRRLASLLVNGNGYPD

SRASQLSAALNYLRTNRPGLDANRLAVAGH**S**MGGGGTLRIAEQNGLPLALLLTPWGGGG

GGGGLLGGG**D**TVAPVSQHAIPFYQNLPGGRLLLVLGG**H**FAPNNGRGGLVLLLLRCQ

> P8

SNCLLLLSLEEALARLAARGLAVALASGYTADASSLAWLGRRLASLGVGVLLLSAGYPDS

RASQLSAALNYLRTNLASRLGLDANRLAVAGH**S**MGGGGTLRIAEQNGVDALLFTPWGGG

GGGG**D**TVAPVSQHAIPFYQNRGLLVVAGN**H**FAPNNGLLLGGLLLLLLRCQ

> P9

SNLAALLSRLGIAIGSGYTADASSLAWLGRRLASLLGVLLVNGNGYPDSRASQLSAALNY

LRTNRPSLDANRLAVAGH**S**MGGGGTLRIAEQNRGGLLAVVFTPWGGGGGGGLLLLLLAG

DTVAPVSQHAIPFYQNGLLLLP**H**FAPNDPADADELLALLLRLLLELLALLEACQ

> P10

SNLALLLLLLRGLALALAAGYTADASSLAWLGRRLASGGVLVVGGGYPDSRASQLSAAL

NYLRTNGLRLAGLPLDANRLAVAGH**S**MGGGGTLRIAEQNPGLDLALVLTPWGGGGSG**D**T

VAPVSQHAIPFYQNRGLLLAAP**H**FAPNNGLALELLLELLAALLGGGGLLLLLLDGGGGV

VLLLLNCQ、

> P4-a

SNAERARALAEVRARGRGALVLDGGYTADASSLAWLGRRLASLFGVLLVNARYPDSRAS
QLSAALNYLRTQLASLGLDANRLAVAGHSMGGGGTLRIAEQNRGRPRVLAFTPWESGPA
RGGRADVLAGRGSDTVAPVSQHAIPFYQNLRGGRLRVLPAPHFAPNRGSPAVVAAARAAL
EAWEAQQ

> P5-a

SNAQLLRAGAQLALVLYAGYTADASSLAWLGRRLASLGLAVLLLSGGGYPDSRASQLSA
ALNYLRTQLASLGLDANRLAVAGHSMGGGGTLRIAEQNRGGLLALAFTPWEGGRAGRAV
LVLGSDTVAPVSQHAIPFYQNARPGGFRLLRVPGNHFAPNNGRALDALEELLQLLQQQQ

> P7-a

SNCSVEVRRLDSLEELADFLEEELGLDDVALASGYTADASSLAWLGRRLASLLVNGNGYP
DSRASQLSAALNYLRTNRPGLDANRLAVAGHSMGGGGTLRIAEQNGLPGALVFTPWGPG
GGGRLLLLGADTVAPVSQHAIPFYQNLPGGRLALSGDGHFAPNNGRGRVLLLEVTCQ

> P4-a-1

SEAALAAAEAQLRALGRGALVVNAGYGESEDALAGLADALADLLGVLRIAAPDPATAAA
DIRAALARLAARLAALGLDPDDLAVLGHSAGGGAALRVAEATPGNPLVISLAPLSPAVARG
GTADIIGGVGADTIAPYERYALPHYAALDGGRLTLLPVPHYAATEGNPEVIAAVRAAIEAW
RAGR

> P4-a-2

TEAEWARALAQLRALGRGALVLNSGYGESPEALAGLGEALSELLGVLLIAAPDPEKAAD
DIEAALARLAAALAALGLDPRLLAVLGHSLGGGATLEVAERHPGWPLVIALAPLAPRVAR
GGTADIIGGVGADTIAPYEEYALPHYAAADGGRLTLLPLPHFAATQGSPAVIAAVKAAVEA
WLATQ

> P5-a-1

MERELLREGGEYSVVIASGYGQTIDSLRGLGEYLASRGLSVLLLSGSGDPETVAEQLAEAL
AELRKRAAALGKDPNRQAVAGHSVGGGGTSVLAARRPDGLLGLAFNPCGGAAARRIILV
TGDDEICPPEKHALPLYAGALPGGAELVEVPGGHFASTVGATREAVDLLLERMAALK

> P5-a-2

MRRELLREGGERGVVLTAGYGQTWRSLRGLGEYLASRGLSVLLLDGEGDPETLAEQIAE

ALAELRARLAALGRDPTRLAVAGH**S**VGGGGTLVLAARRPDGLLALAFRPYGGAAAPRVIL

VTGD**D**TIAPPEENALPLYAGARPGGATLIEVPGD**H**YASTVGRTRAAVDELLARLAALT

Table S3: Changes in the hydrophilic and hydrophobic properties and gyration radii of the proteins after sequence refinement using iterative RF$_{joint}$ and ProteinMPNN.

Protein	Hydrophilic surface area (nm^2)	Hydrophobic surface area (nm^2)	Total surface area (nm^2)	Hydrophobic surface / Total surface	R_g (nm)
LCC	52.7	48.4	101.1	47.7%	1.67
P4	45.0	50.5	95.5	52.9%	1.58
P4-a	46.9	40.7	87.6	46.5%	1.59
P4-a-1	36.9	42.0	78.9	53.3%	1.53
P4-a-2	36.4	43.6	80.0	54.5%	1.55
P5	45.5	41.9	87.4	47.9%	1.51
P5-a	46.9	35.5	82.3	43.1%	1.50
P5-a-1	40.8	39.7	80.5	49.3%	1.49
P5-a-2	43.4	37.9	81.3	46.6%	1.50
P7	45.1	39.3	84.3	46.6%	1.49
P7-a	47.4	37.4	84.8	44.1%	1.53

Table S4. The nucleotide sequences of designer enzymes (codon optimized for *E. coli* expression) used in this study.

All amino acid sequences were optimized using the proprietary codon optimization system developed by BGI Genomics.

> P1

TCTAACACCTTGGGTCTGGGTCTGGAACTGGCTCTGCTTCGTGGTGCTGGTGCTCTTGC
TCTGGCAGGTGGTTACACCGCTGATGCGTCTAGCCTGGCTTGGCTGGGTCGTCGTCTG
GCATCTGAACTGGGTCTTGCTGTTGCTCTGGTTCCAGCTGGTGGTTATCCGGACTCTCG
TGCTTCTCAGCTGTCTGCTGCTCTGAACTACCTGCGTACTCGTCTTGCAGCTCGTGGTC
TGTCTCGTCTGGACGCTAACCGTCTGGCAGTTGCTGGTCACAGCATGGGTGGTGGTGG
TACTCTGCGTATCGCTGAACAGGGTCTGCTTCTGGCTCTGACTCCGTGGGGTGGTGATA
CCGTTGCTCCAGTTTCTCAGCACGCTATTCCGTTCTACCAGAACCTGCTGCGTCTTGGT
CTGCGTGGTGTTGTTCTGGTTGTTGGTGGTAACCACTTCGCTCCGAACAACGGTCTGG
GTTTGTGCCAG

> P2

TCTAACTCTCTGGACGGTCTTGGTGACGCTTTCGCTCTGGCTGCTGGTTACACCGCAGA
TGCGTCTTCTCTGGCTTGGCTGGGTCGTCGTCTGGCTTCTCTGCTGGTTCCAGCTGGTT
ATCCGGACTCTCGTGCTTCTCAGCTGTCTGCTGCGCTGAACTACCTGCGTACTCAGCTG
GCTTCTGAACTGGGTCTGGACGCTAACCGTCTGGCTGTTGCTGGTCACTCTATGGGTG
GTGGTGGTACCTTGCGTATCGCTGAACAGGGTCTGCTGCTTGTTCTGACTCCGTGGGGT
GACACCGTTGCTCCGGTTAGCCAGCACGCTATTCCGTTCTACCAGAACTGCGGTCTTCC
ACTGCTGCTGGCTGGTCCAGGTCACTTCGCTCCGAACAACGGTCTGGGTCTTGGTCTG
AACTGCCAG

> P3

TCTAACGCAGCACTGCTGCTGGCACTTCTGGCTGGTCTGCGTCAAGCTCGTGCTCTTG
CTCTGGCTAGCGGTTACACTGCTGACGCTTCTTCTCTGGCTTGGCTTGGTCGTCGTTTG
GCTTCTGAACTGGGTCTTGGTGTTCTGCTGGTTAACGGTAACGGTTATCCGGACTCTCG

TGCTTCTCAGCTGTCTGCAGCTCTGAACTACCTGCGTACCAACCGTCCAGGTCTGGATG
CTAACCGTCTGGCTGTTGCTGGTCACTCTATGGGTGGCGGTGGTACCTTGCGTATCGCT
GAACAGAACGGTCTGCCAGTAGCTCTGGCTTTCACTCCGTGGGAACCAGGTCCACCTC
GTGGTGGTTTGCTGCTTCTGCTGTTGGGTCTGGGTGGTGGTGATACCGTTGCTCCGGTT
TCTCAGCACGCTATTCCGTTCTACCAGAACTCTGGTCTTGGTCTGGCTTTGCCACACTT
CGCACCGAACAACGGTCGTGCTTTCGAAGCACTGGAAGAACTGCTGGCTCAGTGCCA
G

> P4

TCTAACGCTCTGCTGCTTCTGCTTCTTCTGCTGTTGCTGCTGGGTGGTGGTCTGCTGCT
GGCTCTGGCAGGTGGTTACACCGCTGATGCGAGCTCTCTGGCTTGGCTGGGTCGTCGT
CTGGCTTCTCTGTTCGGTGTTCTGCTGCTGTCTGGTGGTTATCCGGACTCTCGTGCTAG
CCAGCTGTCTGCAGCTCTGAACTACCTGCGTACTCAGCTGGCAAGCCTGGGTCTGGAT
GCGAACCGTCTGGCAGTTGCTGGTCACTCTATGGGTGGTGGTGGTACTCTGCGTATCGC
GGAACAGAACCGTGGCGGTCTGCTGGTTCTGGTGTTCACTCCGTGGGAATCTGGTCCG
GCTCGTGGTGGTCGTCTGGTTGTGCTGGTTGTTGGTGCGTCTGACACCGTTGCTCCGG
TTAGCCAGCATGCGATTCCGTTCTACCAGAACCTGCGTGGTGGTTTGGTTGTTCTGGTT
GGTCTGCCACACTTCGCGCCGAACCGTGGTTCTCCGGAACTGCTGGAAGCACTGCTTC
GTCTGCTGCGTCTGTTGCTGCAGTGCCAG

> P5

TCTAACCTGCTGCTGCTTCTTCTGCTGGGTGCTGGTGCTCTGGCTCTGTACGCGGGTTA
CACCGCTGACGCGTCTTCTCTGGCGTGGCTGGGTCGTCGTCTGGCTAGCCTGGGTCTG
GCGGTACTGCTGCTGTCTGGTGGTGGTTATCCGGACAGTCGTGCTTCTCAGCTGTCTGC
AGCGCTGAACTACCTGCGTACTCAGCTGGCGAGCCTGGGTTTGGACGCGAACCGTCTG
GCTGTTGCGGGTCACAGCATGGGTGGTGGTGGTACCTTGCGTATCGCGGAACAGAACC
GTGGTGGTTTGCTGGCACTGGCGTTCACTCCGTGGGAAGGTGGTCGTGCAGGTCGTGC
TGTTCTGGTTCTGGGTTCTGACACCGTTGCACCGGTTTCTCAGCACGCGATTCCGTTCT
ACCAGAACGCTCGTCCAGGTGGTGGTCTGCTTCTGCTGCTGGGTGGTAACCACTTCGC

TCCGAACAACGGTCGTGCACTGGATGCTCTGGAAGAACTGCTGCAGCTGCTGCAACA

GTGCCAG

> P6

TCTAATCCGCGTCCACGTCTTCTGGCTCTGGCTTCTGGTTACACCGCTGATGCGTCTTCT

CTGGCTTGGTTGGGTCGTCGTTTGGCGTCTCTGTTTCCGAACCTGGGTGTTGCACTGGT

AGATGGCCGTAACGAATATCCGGATTCTCGTGCATCTCAGCTGTCTGCTGCTCTGAACT

ACCTGCGTACTCTGGCTGAAGCTCAAGGTCTGGGTCTGGATGCTAACCGTCTGGCTGT

TGCAGGTCACTCTATGGGTGGTGGTGGTACCTTGCGTATCGCTGAACAGAACGGTGTT

CCGTTCGTTGTGTTCACTCCGTGGGATGCAGAACCACCACGTGGCGGTCGTTTGCTGC

TGGTTGTTGGTGGTCGTAACGACACCGTTGCACCGGTTAGCCAGCATGCGATTCCGTTC

TACCAGAACAATCCGAACCTTCGTCTGGTTCTGCTGCCAGGTAACCACTTCGCTCCGA

ACGATCCGGAGCTGCTGGAAGAACTGCTGGAACTTCTGCTCGAACTGCTGCTGTTGCT

GCCGCAATGCCAG

> P7

TCTAACTGCTCTCTGCTTCTGCTGCTGCTGTCTGACCTGGAAGAGCTGCTGGAACTGCT

GGAAGCTCTGCTGGGTGGTCTGGCTTTCGCTCTGGCATCTGGTTACACCGCTGACGCTT

CTTCTCTGGCTTGGCTGGGTCGTCGTCTGGCTTCTCTGCTGGTTAACGGTAACGGTTAT

CCGGACAGCCGTGCTTCTCAGCTGTCTGCTGCTCTGAACTACCTGCGTACCAACCGTC

CGGGTCTGGACGCAAACCGTCTGGCTGTTGCTGGTCATAGCATGGGTGGTGGCGGTAC

TCTGCGTATCGCTGAACAGAACGGTCTGCCACTGGCACTGCTGCTGACTCCGTGGGGT

GGTGGTGGAGGTGGCGGTGGTCTGCTCGGTGGTGGTGACACCGTTGCACCGGTTTCTC

AGCACGCGATTCCGTTCTACCAGAACCTGCCGGGTGGTCGTTTGCTGCTGGTTCTGGG

TGGTCATTTCGCTCCGAACAACGGTCGTGGTGGTCTGGTGTTGCTGCTGCTTCGTTGCC

AG

> P8

TCTAACTGCCTGCTGCTGCTGTCTCTGGAAGAAGCACTGGCGCGTCTCGCTGCTCGTG

GTCTGGCTGTTGCGCTGGCGTCTGGCTACACTGCGGATGCGTCTTCTCTGGCATGGCTT

GGTCGTCGTCTGGCGTCTCTGGGTGTAGGTGTTCTTCTGCTGTCTGCAGGTTATCCAGA

CAGCCGTGCTTCTCAGCTGTCTGCTGCGCTGAACTACCTGCGTACCAACCTGGCAAGC
CGTCTGGGTCTGGACGCTAACCGTCTGGCAGTTGCGGGTCACTCTATGGGTGGTGGTG
GTACTCTGCGTATCGCGGAACAGAACGGTGTTGACGCTCTGCTGTTCACTCCGTGGGG
TGGTGGTGGCGGTGGTGGTGACACTGTTGCGCCAGTTTCTCAGCACGCGATTCCGTTC
TACCAGAACCGTGGTCTGCTGGTTGTTGCTGGTAACCACTTCGCTCCGAACAACGGTC
TGCTGCTGGGTGGTTTGCTGTTGCTTCTGCTGCGTTGCCAG

> P9

TCTAACCTGGCTGCACTGCTGTCTCGTCTGGGTATCGCTATCGGTTCTGGTTACACCGC
AGACGCATCTAGCCTGGCTTGGCTGGGTCGTCGTCTGGCTAGCCTGCTGGGTGTTCTG
CTGGTTAACGGTAACGGTTATCCGGATTCTCGTGCTAGCCAGCTGTCTGCAGCACTGAA
CTACCTGCGTACCAACCGTCCGTCTCTGGATGCGAACCGTCTGGCGGTTGCAGGTCAC
TCTATGGGTGGTGGCGGTACTCTGCGTATCGCGGAACAGAACCGTGGTGGTCTTCTGG
CTGTTGTGTTCACTCCGTGGGGTGGCGGTGGTGGTGGTGGTCTGTTGCTGCTGCTGTT
GGCTGGTGACACCGTTGCTCCGGTTTCTCAGCACGCGATTCCGTTCTACCAGAACGGT
CTGCTTCTGCTGCCGCACTTCGCACCGAACGATCCAGCGGATGCGGATGAACTGCTGG
CACTGCTGCTGCGTCTTCTGCTGGAACTTCTGGCTCTGCTGGAGGCTTGCCAG

> P10

TCTAACCTGGCACTGCTGCTGTTGCTTCTGCGTGGTCTGGCACTTGCTCTGGCTGCTGG
TTACACCGCAGATGCGTCTTCTCTGGCTTGGCTGGGTCGTCGTCTGGCTTCTGGTGGTG
TTCTGGTTGTTGGTGGTGGTTATCCGGACTCTCGTGCTTCTCAGCTGTCTGCTGCTCTG
AACTACCTGCGTACTAACGGTCTGCGTCTTGCTGGTCTGCCACTGGACGCTAACCGTCT
GGCTGTTGCTGGTCACTCTATGGGTGGTGGCGGTACTCTGCGTATCGCGGAACAGAATC
CGGGTCTGGATCTGGCTCTGGTTCTGACTCCGTGGGGTGGCGGTGGTTCTGGTGACAC
CGTTGCTCCGGTTTCTCAGCACGCGATTCCGTTCTACCAGAACCGTGGTCTTCTGCTGG
CAGCTCCGCACTTCGCGCCGAACAACGGTCTGGCGCTGGAACTGCTGCTTGAACTGCT
GGCTGCTCTGCTGGGTGGAGGTGGTCTGCTTCTGCTGCTGCTTCTGGACGGTGGTGGT
GGCGTTGTTCTGCTCTTGCTGAACTGCCAG

> P4-a

TCTAACGCGGAACGTGCTCGTGCTCTGGCAGAAGTTCGTGCACGTGGTCGTGGTGCTC
TGGTACTGGACGGTGGTTACACCGCGGATGCAAGCTCTCTGGCATGGTTGGGTCGTCG
TCTGGCATCTCTGTTCGGTGTTCTGCTGGTTAACGCACGTTATCCGGATAGCCGTGCAA
GCCAGCTGTCCGCTGCTCTGAACTACCTGCGTACTCAGCTGGCTAGCCTGGGTCTGGAT
GCAAACCGTCTGGCAGTTGCAGGTCACAGCATGGGTGGTGGTGGTACTCTGCGTATCG
CTGAACAGAACCGTGGTCGTCCACGTGTACTGGCATTTACTCCGTGGGAATCTGGTCC
GGCTCGTGGTGGCCGTGCGGATGTACTGGCGGGTCGTGGTTCTGACACCGTTGCTCCG
GTTTCTCAGCACGCAATTCCGTTCTACCAGAACCTTCGTGGTGGTCGTCTGCGTGTTCT
GCCAGCACCGCACTTCGCACCAAACCGTGGTAGTCCAGCGGTTGTAGCTGCTGCTCGT
GCAGCACTGGAAGCTTGGGAAGCTCAGCAG

> P5-a

TCTAACGCGCAGCTGCTGCGTGCTGGTGCTCAGCTGGCTCTGGTACTGTACGCTGGTTA
CACTGCGGACGCTAGCTCTCTGGCTTGGCTGGGTCGTCGTCTGGCGTCTCTCGGTCTG
GCAGTTCTTCTGCTGTCTGGTGGTGGTTATCCGGACTCTCGTGCGTCTCAGCTGTCTGC
TGCTCTGAACTACCTGCGTACTCAACTGGCTTCTCTGGGTCTGGACGCTAACCGTCTGG
CGGTTGCTGGTCACAGCATGGGTGGTGGTGGTACTCTGCGTATCGCGGAACAGAACCG
TGGTGGTCTGCTGGCGCTGGCGTTCACGCCTTGGGAAGGTGGTCGTGCTGGTCGTGCA
GTTCTGGTTCTGGGTTCTGACACCGTTGCGCCGGTAAGCCAGCACGCGATTCCGTTCTA
CCAGAACGCACGTCCAGGTGGTTTCCGTCTGCTTCGTGTTCCGGGTAACCACTTCGCT
CCAAACAACGGTCGTGCTCTGGACGCACTGGAAGAACTGCTGCAGTTGCTGCAACAG
CAGCAG

> P7-a

TCCAACTGTTCTGTAGAAGTACGTCGTCTGGACTCTCTGGAAGAACTGGCGGACTTTC
TGGAAGAAGAACTGGGTCTGGATGACGTAGCTCTGGCAAGCGGTTACACCGCTGATGC
GTCTTCTCTGGCTTGGCTGGGTCGTCGTCTTGCGTCTCTGCTGGTTAACGGCAACGGTT
ATCCAGACTCTCGTGCGTCTCAGCTGTCTGCGGCTCTGAACTACCTGCGTACCAACCGT
CCAGGTCTGGACGCAAACCGTCTGGCTGTTGCAGGTCACTCTATGGGTGGTGGTGGTA

CTCTGCGTATCGCAGAACAGAACGGTCTGCCAGGTGCTCTGGTATTCACTCCGTGGGG
TCCAGGTGGTGGCGGTCGTCTGCTGCTGCTGGGCGCTGATACCGTTGCGCCAGTTAGC
CAGCATGCGATTCCGTTCTATCAGAACCTGCCGGGTGGTCGTCTGGCTCTGTCTGGTGA
TGGTCACTTCGCGCCAAACAACGGTCGTGGTCGTGTACTTCTGCTGGAAGTGACCTGC
CAG

> P4-a-1

AGCGAAGCAGCACTGGCAGCAGCAGAAGCACAGCTGCGTGCATTAGGTCGTGGTGCA
TTAGTTGTTAATGCAGGTTATGGTGAAAGCGAGGATGCACTGGCAGGTCTGGCAGATG
CACTGGCAGATCTGCTGGGTGTTCTGCGTATTGCAGCACCGGACCCGGCAACCGCAGC
AGCAGATATTCGTGCAGCACTGGCACGTCTGGCAGCACGTTTAGCAGCACTGGGTTTA
GATCCGGATGATCTGGCAGTTCTGGGTCATAGCGCAGGTGGTGGTGCAGCATTACGTGT
TGCAGAAGCAACCCCGGGTAATCCGCTGGTTATTAGCCTGGCACCGCTGAGCCCTGCA
GTTGCACGTGGTGGTACAGCAGATATTATTGGTGGTGTTGGTGCAGATACCATTGCACC
GTATGAACGTTATGCACTGCCGCATTATGCAGCACTGGATGGTGGTCGTCTGACCCTGT
TACCTGTTCCGCATTATGCAGCAACCGAAGGTAATCCGGAAGTTATTGCAGCAGTTCGT
GCAGCAATTGAAGCATGGCGTGCAGGTCGT

> P4-a-2

ACCGAAGCAGAATGGGCACGTGCACTGGCACAGCTGCGTGCATTAGGTCGTGGTGCA
CTGGTTTTAAATAGCGGTTATGGTGAAAGCCCGGAAGCACTGGCAGGTCTGGGTGAAG
CATTAAGCGAACTGCTGGGTGTTCTGCTGATTGCAGCACCTGATCCGGAAAAAGCAGC
AGATGATATTGAAGCAGCACTGGCACGTCTGGCAGCAGCATTAGCAGCATTAGGTTTA
GATCCTCGTTTACTGGCAGTTCTGGGTCATAGCCTGGGTGGTGGTGCAACCTTAGAAGT
TGCAGAACGTCATCCGGGTTGGCCGCTGGTTATTGCACTGGCACCTCTGGCACCGCGT
GTTGCACGTGGTGGTACAGCAGATATTATTGGTGGTGTTGGTGCAGATACCATTGCACC
GTATGAAGAATATGCACTGCCGCATTATGCAGCAGCAGATGGTGGTCGTCTGACCCTGT
TACCGCTGCCGCATTTTGCAGCAACACAGGGTAGCCCGGCAGTTATTGCAGCAGTTAA
AGCAGCAGTTGAAGCATGGCTGGCAACCCAG

> P5-a-1

ATGGAACGTGAACTGCTGCGTGAAGGTGGTGAATATAGCGTTGTTATTGCAAGCGGTT
ATGGCCAGACCATTGATAGCCTGCGTGGTCTGGGTGAATATCTGGCAAGCCGTGGTCTG
AGCGTTCTGCTGTTAAGTGGTAGCGGTGATCCGGAAACAGTTGCAGAACAGCTGGCA
GAAGCACTGGCAGAACTGCGTAAACGTGCAGCAGCACTGGGTAAAGATCCGAATCGT
CAGGCAGTTGCAGGTCATAGCGTTGGTGGTGGTGGTACCAGCGTTCTGGCAGCACGTC
GTCCTGATGGTTTACTGGGTCTGGCATTTAATCCGTGTGGTGGTGCAGCAGCACGTCGT
ATTATTCTGGTTACCGGTGATGATGAAATTTGCCCGCCGGAAAAACATGCACTGCCGCT
GTATGCAGGTGCACTGCCGGGTGGTGCAGAACTGGTTGAAGTTCCGGGTGGTCATTTT
GCAAGCACCGTTGGTGCAACCCGTGAAGCAGTTGATCTGCTGCTGGAACGTATGGCAG
CACTGAAA

> P5-a-2

ATGCGTCGTGAACTGCTGCGTGAAGGTGGTGAACGTGGTGTTGTTCTGACCGCAGGTT
ATGGTCAGACCTGGCGTAGCCTGCGTGGTCTGGGTGAATATCTGGCAAGCCGTGGTCT
GAGCGTTCTGCTGTTAGATGGTGAAGGTGATCCGGAAACCCTGGCAGAACAGATTGCA
GAAGCACTGGCAGAACTGCGTGCACGTCTGGCAGCATTAGGTCGTGATCCGACCCGTC
TGGCAGTTGCAGGTCATAGCGTTGGTGGTGGTGGTACCCTGGTTCTGGCAGCACGTCG
TCCTGATGGTCTGCTGGCATTAGCATTTCGTCCGTATGGTGGTGCAGCAGCACCGCGTG
TTATTCTGGTTACCGGTGATGATACCATTGCACCGCCGGAAGAAAATGCACTGCCGCTG
TATGCAGGTGCACGTCCGGGTGGTGCAACACTGATTGAAGTTCCGGGTGATCATTATGC
AAGCACCGTTGGTCGTACCCGTGCAGCAGTTGATGAACTGCTGGCACGTCTGGCAGCA
CTGACC

Table S5: Vectors and bacterial stains used for enzyme expression in this study.

Protein	Vector	Strain
P1-P10	pET28a (+)	*E. Coli* BL21(DE3)
P4-a， P5-a and P7-a	pET28a (+)	*E. Coli* Lemo21 (DE3)
SUMO-tagged P4-a and P5-a	pET28a (+)	*E. Coli* BL21 (DE3) PLysS
P4-a-1， P4-a-2， P5-a-1 and P5-a-2	pET29b (+)	*E. Coli* BL21(DE3)

Table S6: Primers used in this study

1. Primers for pET28a/His-SUMO A vector linearization

Forward primer: 5'- CTCGAGTTATTATGCCCTTGAAGGC -3'

Reverse primer: 5'- GGATCCACCAATCTGTTCTCTGTGA -3'

2. Primers for P4-a

Forward primer: 5'- gggaaaGGATCCTCTAACGCGGAACGT -3'

Reverse primer: 5'- gggaaaCTCGAGTTACTGCTGAGCTTCCCA -3'

3. Primers for P5-a

Forward primer: 5'- gggaaaGGATCCTCTAACGCGCAGCT -3'

Reverse primer: 5'- gggaaaCTCGAGTTACTGCTGCTGTTGCAG -3'

4. Primers for DNA sequencing

His-SUMO-P4-a

5'- CCATACCCACGCCGAAACAAG -3' (pET28a Forward, a universal primer)

5'- GCGTACTCAGCTGGCTAGCC -3'

5'- CGTCAAAGGGCGAAAAACCGT -3'

His-SUMO-P5-a

5'- CCATACCCACGCCGAAACAAG -3' (pET28a Forward, a universal primer)

5'- GGCTTCTCTGGGTCTGGACG -3'

5'- ACCGTCTATCAGGGCGATGG-3'

5. DNA sequences

Start and stop codons for His-SUMO-P4-a/P5-a are highlighted in blue.

His tag is labeled orange.

SUMO tag is labeled red.

The sequences of P4-a/P5-a enzyme are labeled green, with the cleavage sites underlined.

> pET28a/His-SUMO vector

CTCGAGTTATTATGCCCTTGAAGGCTTGTAGGTCTGGGAGGCGAGGGTCTGGCAGTGA

GGCGCCTGCAACGGCGGGTGTGGTGGTTACGCGCAGCGTGACCGCTACACTTGCCAG
CGCCCTAGCGCCCGCTCCTTTCGCTTTCTTCCCTTCCTTTCTCGCCACGTTCGCCGGCTT
TCCCCGTCAAGCTCTAAATCGGGGGCTCCCTTTAGGGTTCCGATTTAGTGCTTTACGGC
ACCTCGACCCCAAAAAACTTGATTAGGGTGATGGTTCACGTAGTGGGCCATCGCCCTG
ATAGACGGTTTTTCGCCCTTTGACGTTGGAGTCCACGTTCTTTAATAGTGGACTCTTGTT
CCAAACTGGAACAACACTCAACCCTATCTCGGTCTATTCTTTTGATTTATAAGGGATTTT
GCCGATTTCGGCCTATTGGTTAAAAAATGAGCTGATTTAACAAAAATTTAACGCGAATT
TTAACAAAATATTAACGTTTACAATTTCAGGTGGCACTTTTCGGGGAAATGTGCGCGGA
ACCCCTATTTGTTTATTTTTCTAAATACATTCAAATATGTATCCGCTCATGAATTAATTCTT
AGAAAAACTCATCGAGCATCAAATGAAACTGCAATTTATTCATATCAGGATTATCAATAC
CATATTTTTGAAAAAGCCGTTTCTGTAATGAAGGAGAAAACTCACCGAGGCAGTTCCA
TAGGATGGCAAGATCCTGGTATCGGTCTGCGATTCCGACTCGTCCAACATCAATACAAC
CTATTAATTTCCCCTCGTCAAAAATAAGGTTATCAAGTGAGAAATCACCATGAGTGACG
ACTGAATCCGGTGAGAATGGCAAAAGTTTATGCATTTCTTTCCAGACTTGTTCAACAGG
CCAGCCATTACGCTCGTCATCAAAATCACTCGCATCAACCAAACCGTTATTCATTCGTG
ATTGCGCCTGAGCGAGACGAAATACGCGATCGCTGTTAAAAGGACAATTACAAACAGG
AATCGAATGCAACCGGCGCAGGAACACTGCCAGCGCATCAACAATATTTTCACCTGAA
TCAGGATATTCTTCTAATACCTGGAATGCTGTTTTCCCGGGGATCGCAGTGGTGAGTAA
CCATGCATCATCAGGAGTACGGATAAAATGCTTGATGGTCGGAAGAGGCATAAATTCCG
TCAGCCAGTTTAGTCTGACCATCTCATCTGTAACATCATTGGCAACGCTACCTTTGCCAT
GTTTCAGAAACAACTCTGGCGCATCGGGCTTCCCATACAATCGATAGATTGTCGCACCT
GATTGCCCGACATTATCGCGAGCCCATTTATACCCATATAAATCAGCATCCATGTTGGAA
TTTAATCGCGGCCTAGAGCAAGACGTTTCCCGTTGAATATGGCTCATAACACCCCTTGT
ATTACTGTTTATGTAAGCAGACAGTTTTATTGTTCATGACCAAAATCCCTTAACGTGAGT
TTTCGTTCCACTGAGCGTCAGACCCCGTAGAAAAGATCAAAGGATCTTCTTGAGATCC
TTTTTTTCTGCGCGTAATCTGCTGCTTGCAAACAAAAAAACCACCGCTACCAGCGGTG
GTTTGTTTGCCGGATCAAGAGCTACCAACTCTTTTTCCGAAGGTAACTGGCTTCAGCA
GAGCGCAGATACCAAATACTGTCCTTCTAGTGTAGCCGTAGTTAGGCCACCACTTCAAG
AACTCTGTAGCACCGCCTACATACCTCGCTCTGCTAATCCTGTTACCAGTGGCTGCTGC
CAGTGGCGATAAGTCGTGTCTTACCGGGTTGGACTCAAGACGATAGTTACCGGATAAG
GCGCAGCGGTCGGGCTGAACGGGGGGTTCGTGCACACAGCCCAGCTTGGAGCGAACG

ACCTACACCGAACTGAGATACCTACAGCGTGAGCTATGAGAAAGCGCCACGCTTCCCG
AAGGGAGAAAGGCGGACAGGTATCCGGTAAGCGGCAGGGTCGGAACAGGAGAGCGC
ACGAGGGAGCTTCCAGGGGGAAACGCCTGGTATCTTTATAGTCCTGTCGGGTTTCGCC
ACCTCTGACTTGAGCGTCGATTTTTGTGATGCTCGTCAGGGGGGCGGAGCCTATGGAA
AAACGCCAGCAACGCGGCCTTTTTACGGTTCCTGGCCTTTTGCTGGCCTTTTGCTCACA
TGTTCTTTCCTGCGTTATCCCCTGATTCTGTGGATAACCGTATTACCGCCTTTGAGTGAG
CTGATACCGCTCGCCGCAGCCGAACGACCGAGCGCAGCGAGTCAGTGAGCGAGGAAG
CGGAAGAGCGCCTGATGCGGTATTTTCTCCTTACGCATCTGTGCGGTATTTCACACCGC
ATATATGGTGCACTCTCAGTACAATCTGCTCTGATGCCGCATAGTTAAGCCAGTATACAC
TCCGCTATCGCTACGTGACTGGGTCATGGCTGCGCCCCGACACCCGCCAACACCCGCT
GACGCGCCCTGACGGGCTTGTCTGCTCCCGGCATCCGCTTACAGACAAGCTGTGACCG
TCTCCGGGAGCTGCATGTGTCAGAGGTTTTCACCGTCATCACCGAAACGCGCGAGGCA
GCTGCGGTAAAGCTCATCAGCGTGGTCGTGAAGCGATTCACAGATGTCTGCCTGTTCAT
CCGCGTCCAGCTCGTTGAGTTTCTCCAGAAGCGTTAATGTCTGGCTTCTGATAAAGCGG
GCCATGTTAAGGGCGGTTTTTTCCTGTTTGGTCACTGATGCCTCCGTGTAAGGGGGATT
TCTGTTCATGGGGGTAATGATACCGATGAAACGAGAGAGGATGCTCACGATACGGGTT
ACTGATGATGAACATGCCCGGTTACTGGAACGTTGTGAGGGTAAACAACTGGCGGTAT
GGATGCGGCGGGACCAGAGAAAAATCACTCAGGGTCAATGCCAGCGCTTCGTTAATAC
AGATGTAGGTGTTCCACAGGGTAGCCAGCAGCATCCTGCGATGCAGATCCGGAACATA
ATGGTGCAGGGCGCTGACTTCCGCGTTTCCAGACTTTACGAAACACGGAAACCGAAG
ACCATTCATGTTGTTGCTCAGGTCGCAGACGTTTTGCAGCAGCAGTCGCTTCACGTTCG
CTCGCGTATCGGTGATTCATTCTGCTAACCAGTAAGGCAACCCCGCCAGCCTAGCCGGG
TCCTCAACGACAGGAGCACGATCATGCGCACCCGTGGGGCCGCCATGCCGGCGATAAT
GGCCTGCTTCTCGCCGAAACGTTTGGTGGCGGGACCAGTGACGAAGGCTTGAGCGAG
GGCGTGCAAGATTCCGAATACCGCAAGCGACAGGCCGATCATCGTCGCGCTCCAGCGA
AAGCGGTCCTCGCCGAAAATGACCCAGAGCGCTGCCGGCACCTGTCCTACGAGTTGC
ATGATAAAGAAGACAGTCATAAGTGCGGCGACGATAGTCATGCCCCGCGCCCACCGGA
AGGAGCTGACTGGGTTGAAGGCTCTCAAGGGCATCGGTCGAGATCCCGGTGCCTAATG
AGTGAGCTAACTTACATTAATTGCGTTGCGCTCACTGCCCGCTTTCCAGTCGGGAAACC
TGTCGTGCCAGCTGCATTAATGAATCGGCCAACGCGCGGGGAGAGGCGGTTTGCGTAT
TGGGCGCCAGGGTGGTTTTTCTTTTCACCAGTGAGACGGGCAACAGCTGATTGCCCTT

CACCGCCTGGCCCTGAGAGAGTTGCAGCAAGCGGTCCACGCTGGTTTGCCCCAGCAG

GCGAAAATCCTGTTTGATGGTGGTTAACGGCGGGATATAACATGAGCTGTCTTCGGTAT

CGTCGTATCCCACTACCGAGATATCCGCACCAACGCGCAGCCCGGACTCGGTAATGGC

GCGCATTGCGCCCAGCGCCATCTGATCGTTGGCAACCAGCATCGCAGTGGGAACGATG

CCCTCATTCAGCATTTGCATGGTTTGTTGAAAACCGGACATGGCACTCCAGTCGCCTTC

CCGTTCCGCTATCGGCTGAATTTGATTGCGAGTGAGATATTTATGCCAGCCAGCCAGAC

GCAGACGCGCCGAGACAGAACTTAATGGGCCCGCTAACAGCGCGATTTGCTGGTGAC

CCAATGCGACCAGATGCTCCACGCCCAGTCGCGTACCGTCTTCATGGGAGAAAATAAT

ACTGTTGATGGGTGTCTGGTCAGAGACATCAAGAAATAACGCCGGAACATTAGTGCAG

GCAGCTTCCACAGCAATGGCATCCTGGTCATCCAGCGGATAGTTAATGATCAGCCCACT

GACGCGTTGCGCGAGAAGATTGTGCACCGCCGCTTTACAGGCTTCGACGCCGCTTCGT

TCTACCATCGACACCACCACGCTGGCACCCAGTTGATCGGCGCGAGATTTAATCGCCG

CGACAATTTGCGACGGCGCGTGCAGGGCCAGACTGGAGGTGGCAACGCCAATCAGCA

ACGACTGTTTGCCCGCCAGTTGTTGTGCCACGCGGTTGGGAATGTAATTCAGCTCCGC

CATCGCCGCTTCCACTTTTTCCCGCGTTTTCGCAGAAACGTGGCTGGCCTGGTTCACCA

CGCGGGAAACGGTCTGATAAGAGACACCGGCATACTCTGCGACATCGTATAACGTTAC

TGGTTTCACATTCACCACCCTGAATTGACTCTCTTCCGGGCGCTATCATGCCATACCGC

GAAAGGTTTTGCGCCATTCGATGGTGTCCGGGATCTCGACGCTCTCCCTTATGCGACTC

CTGCATTAGGAAGCAGCCCAGTAGTAGGTTGAGGCCGTTGAGCACCGCCGCCGCAAG

GAATGGTGCATGCAAGGAGATGGCGCCCAACAGTCCCCCGGCCACGGGGCCTGCCAC

CATACCCACGCCGAAACAAGCGCTCATGAGCCCGAAGTGGCGAGCCCGATCTTCCCCA

TCGGTGATGTCGGCGATATAGGCGCCAGCAACCGCACCTGTGGCGCCGGTGATGCCGG

CCACGATGCGTCCGGCGTAGAGGATCGAGATCTCGATCCCGCGAAATTAATACGACTCA

CTATAGGGGAATTGTGAGCGGATAACAATTCCCCTCTAGAAATAATTTTGTTTAACTTTA

AGAAGGAGATATACC**ATG**GGCAGCAGCCATCATCATCATCATCACAGCAGCGGCCTGG

TGCCGCGCGGCAGCCATATGGCTAGCATGTCGGACTCAGAAGTCAATCAAGAAGCTAA

GCCAGAGGTCAAGCCAGAAGTCAAGCCTGAGACTCACATCAATTTAAAGGTGTCCGAT

GGATCTTCAGAGATCTTCTTCAAGATCAAAAAGACCACTCCTTTAAGAAGGCTGATGG

AAGCGTTCGCTAAAAGACAGGGTAAGGAAATGGACTCCTTAAGATTCTTGTACGACGG

TATTAGAATTCAAGCTGATCAGACCCCTGAAGATTTGGACATGGAGGATAACGATATTAT

TGAGGCTCACAGAGAACAGATTGGTGGATCC

> His-SUMO-P4-a

ATGGGCAGCAGCCATCATCATCATCATCACAGCAGCGGCCTGGTGCCGCGCGGCAGCC

ATATGGCTAGCATGTCGGACTCAGAAGTCAATCAAGAAGCTAAGCCAGAGGTCAAGCC

AGAAGTCAAGCCTGAGACTCACATCAATTTAAAGGTGTCCGATGGATCTTCAGAGATC

TTCTTCAAGATCAAAAAGACCACTCCTTTAAGAAGGCTGATGGAAGCGTTCGCTAAAA

GACAGGGTAAGGAAATGGACTCCTTAAGATTCTTGTACGACGGTATTAGAATTCAAGCT

GATCAGACCCCTGAAGATTTGGACATGGAGGATAACGATATTATTGAGGCTCACAGAG

AACAGATTGGTGGATCCTCTAACGCGGAACGTGCTCGTGCTCTGGCAGAAGTTCGTGC

ACGTGGTCGTGGTGCTCTGGTACTGGACGGTGGTTACACCGCGGATGCAAGCTCTCTG

GCATGGTTGGGTCGTCGTCTGGCATCTCTGTTCGGTGTTCTGCTGGTTAACGCACGTTA

TCCGGATAGCCGTGCAAGCCAGCTGTCCGCTGCTCTGAACTACCTGCGTACTCAGCTG

GCTAGCCTGGGTCTGGATGCAAACCGTCTGGCAGTTGCAGGTCACAGCATGGGTGGTG

GTGGTACTCTGCGTATCGCTGAACAGAACCGTGGTCGTCCACGTGTACTGGCATTTACT

CCGTGGGAATCTGGTCCGGCTCGTGGTGGCCGTGCGGATGTACTGGCGGGTCGTGGTT

CTGACACCGTTGCTCCGGTTTCTCAGCACGCAATTCCGTTCTACCAGAACCTTCGTGGT

GGTCGTCTGCGTGTTCTGCCAGCACCGCACTTCGCACCAAACCGTGGTAGTCCAGCGG

TTGTAGCTGCTGCTCGTGCAGCACTGGAAGCTTGGGAAGCTCAGCAG**TAA**

> His-SUMO-P5-a

ATGGGCAGCAGCCATCATCATCATCATCACAGCAGCGGCCTGGTGCCGCGCGGCAGCC

ATATGGCTAGCATGTCGGACTCAGAAGTCAATCAAGAAGCTAAGCCAGAGGTCAAGCC

AGAAGTCAAGCCTGAGACTCACATCAATTTAAAGGTGTCCGATGGATCTTCAGAGATC

TTCTTCAAGATCAAAAAGACCACTCCTTTAAGAAGGCTGATGGAAGCGTTCGCTAAAA

GACAGGGTAAGGAAATGGACTCCTTAAGATTCTTGTACGACGGTATTAGAATTCAAGCT

GATCAGACCCCTGAAGATTTGGACATGGAGGATAACGATATTATTGAGGCTCACAGAG

AACAGATTGGTGGATCCTCTAACGCGCAGCTGCTGCGTGCTGGTGCTCAGCTGGCTCT

GGTACTGTACGCTGGTTACACTGCGGACGCTAGCTCTCTGGCTTGGCTGGGTCGTCGTC

TGGCGTCTCTCGGTCTGGCAGTTCTTCTGCTGTCTGGTGGTGGTTATCCGGACTCTCGT

GCGTCTCAGCTGTCTGCTGCTCTGAACTACCTGCGTACTCAACTGGCTTCTCTGGGTCT

GGACGCTAACCGTCTGGCGGTTGCTGGTCACAGCATGGGTGGTGGTGGTACTCTGCGT

ATCGCGGAACAGAACCGTGGTGGTCTGCTGGCGCTGGCGTTCACGCCTTGGGAAGGT

GGTCGTGCTGGTCGTGCAGTTCTGGTTCTGGGTTCTGACACCGTTGCGCCGGTAAGCC

AGCACGCGATTCCGTTCTACCAGAACGCACGTCCAGGTGGTTTCCGTCTGCTTCGTGTT

CCGGGTAACCACTTCGCTCCAAACAACGGTCGTGCTCTGGACGCACTGGAAGAACTG

CTGCAGTTGCTGCAACAGCAGCAG**TAA**CTCGAG

6. Protein sequences

Start and stop codons for His-SUMO-P4-a/P5-a are highlighted in blue.

His tag is labeled orange.

SUMO tag is labeled red.

The sequences of P4-a/P5-a enzyme are labeled green, with cleavage sites underlined.

> pET28a/His-SUMO vector

MGSSHHHHHHSQGLLALGTPLQWFESRTYNEHIRDEGIEQLLYIFQAAGKRDNDPLFWGD

ELEYMVVDFDDKERNSMLDVCHDKILTELNMEDSSLCEANDVSFHPEYGRYMLEATPAS

PYLNYVGSYVEVNMQKRRAIAEYKLSEYARQDSKNNLHVGSRSVPLTLTVFPRMGCPDFI

NIKDPWNHKNAASRSLFLPDEVINRHVRFPNLTASIRTRRGEKVCMNVPMYKDIATPETDD

SIYDRDWFLPEDKEAKLASKPGFIYMDSMGFGMGCSCLQVTFQAPNINKARYLYDALVNF

APIMLAFSAAAPAFKGWLADQDVRWNVISGAVDDRTPKERGVAPLLPKYNKNGFGGIAK

DVQDKVLEIPKSRYSSVDLFLGGSKFFNRTYNDTNVPINEKVLGRLLENDKAPLDYDLAK

HFAHLYIRDPVSTFEELLNQDNKTSSNHFENIQSTNWQTLRFKPPTQQATPDKKDSPGWRV

EFRPFEVQLLDFENAAYSVLIYLIVDSILTFSDNINAYIHMSKVWENMKIAHHRDAILFEKF

HWKKSFRNDTDVETEDYSISEIFHNPENGIFPQFVTPILCQKGFVTKDWKELKHSSKHERL

YYYLKLISDRASGELPTTAKFFRNFVLQHPDYKHDSKISKSINYDLLSTCDRLTHLDDSKG

ELTSFLGAEIAEYVKKNKPSIESKCGAAGENLYFQSGAAGTRLSERLTLKPRGKQISSAPHA

DQPITGDVSAANKDAIRKQMDAAASKGDVETYRKLKAKLKGIR

> His-SUMO-P4-a

HHHHHHSSGLVPRGSHMASMSDSEVNQEAKPEVKPEVKPETHINLKVSDGSSEIFFKIKKT

TPLRRLMEAFAKRQGKEMDSLRFLYDGIRIQADQTPEDLDMEDNDIIEAHREQIGGSSNAE

RARALAEVRARGRGALVLDGGYTADASSLAWLGRRLASLFGVLLVNARYPDSRASQLSA

ALNYLRTQLASLGLDANRLAVAGHSMGGGGTLRIAEQNRGRPRVLAFTPWESGPARGGR

ADVLAGRGSDTVAPVSQHAIPFYQNLRGGRLRVLPAPHFAPNRGSPAVVAAARAALEAWE

AQQ

> His-SUMO-P5-a

HHHHHHSSGLVPRGSHMASMSDSEVNQEAKPEVKPEVKPETHINLKVSDGSSEIFFKIKKT

TPLRRLMEAFAKRQGKEMDSLRFLYDGIRIQADQTPEDLDMEDNDIIEAHREQIGGSSNAQ

LLRAGAQLALVLYAGYTADASSLAWLGRRLASLGLAVLLLSGGGYPDSRASQLSAALNYL

RTQLASLGLDANRLAVAGHSMGGGGTLRIAEQNRGGLLALAFTPWEGGRAGRAVLVLGS

DTVAPVSQHAIPFYQNARPGGFRLLRVPGNHFAPNNGRALDALEELLQLLQQQQ

Table S7: Michaelis-Menten kinetic parameters of LCC and *Rs*PETase 1 using PET microparticles as the substrate

	K_m (g/L)	V_{max} (μM/min^{-1})	k_{cat} (min^{-1})	k_{cat}/K_m (L/g/min)
LCC	3.96±0.27	2.78±0.06	106.9	27.0
*Rs*PETase 1	0.72±0.05	0.55±0.01	18.3	25.5

References:

[1] J. A. Lemkul, *Living Journal of Computational Molecular Science* **2019**, *1*.

[2] J. Huang, S. Rauscher, G. Nawrocki, T. Ran, M. Feig, B. L. de Groot, H. Grubmüller, A. D. MacKerell, *Biophys. J.* **2017**, *112*, 175a–176a.

[3] A. K. Mehta, U. K. Gaur, B. Wunderlich, *J. Polym. Sci. Polym. Phys. Ed.* **1978**, *16*, 289–296.

[4] L. Aristizábal-Lanza,; S. V. Mankar, C. Tullberg, B. Zhang, J. A. Linares-Pastén, *Frontiers in Chemical Engineering*. **2022**, *4*.

[5] F. Madeira, M. Pearce, A. R. N. Tivey, P. Basutkar, J. Lee, O. Edbali, N. Madhusoodanan, A. Kolesnikov, R. Lopez, *Nucleic Acids Res*. **2022**, *50*, W276–W279.

[6] A. Stivala, M. Wybrow, A. Wirth, J. C. Whisstock, P. J. Stuckey, *Bioinformatics.* **2011**, *27*, 3315–3316.

[7] F. Flachsenberg, A. Meyder, K. Sommer, P. Penner, M. Rarey, *J. Chem. Inf. Model.* **2020**, *60*, 6502–6522.
